# H3K9 Methyltransferases Suv39h1 and Suv39h2 Control the Differentiation of Neural Progenitor Cells in the Adult Hippocampus

**DOI:** 10.3389/fcell.2021.778345

**Published:** 2022-01-12

**Authors:** Miguel V. Guerra, Matías I. Cáceres, Andrea Herrera-Soto, Sebastián B. Arredondo, Manuel Varas-Godoy, Brigitte van Zundert, Lorena Varela-Nallar

**Affiliations:** ^1^ Institute of Biomedical Sciences, Faculty of Medicine and Faculty of Life Sciences, Universidad Andres Bello, Santiago, Chile; ^2^ Cancer Cell Biology Lab, Centro de Biología Celular y Biomedicina (CEBICEM), Facultad de Medicina y Ciencia, Universidad San Sebastián, Santiago, Chile; ^3^ Centro de Envejecimiento y Regeneración (CARE-UC), Facultad de Ciencias Biológicas, P. Universidad Católica de Chile, Santiago, Chile

**Keywords:** adult neurogenesis, differentiation, epigenetics, H3K9me3, Suv39h1, Suv39h2

## Abstract

In the dentate gyrus of the adult hippocampus new neurons are generated from neural precursor cells through different stages including proliferation and differentiation of neural progenitor cells and maturation of newborn neurons. These stages are controlled by the expression of specific transcription factors and epigenetic mechanisms, which together orchestrate the progression of the neurogenic process. However, little is known about the involvement of histone posttranslational modifications, a crucial epigenetic mechanism in embryonic neurogenesis that regulates fate commitment and neuronal differentiation. During embryonic development, the repressive modification trimethylation of histone H3 on lysine 9 (H3K9me3) contributes to the cellular identity of different cell-types. However, the role of this modification and its H3K9 methyltransferases has not been elucidated in adult hippocampal neurogenesis. We determined that during the stages of neurogenesis in the adult mouse dentate gyrus and in cultured adult hippocampal progenitors (AHPs), there was a dynamic change in the expression and distribution of H3K9me3, being enriched at early stages of the neurogenic process. A similar pattern was observed in the hippocampus for the dimethylation of histone H3 on lysine 9 (H3K9me2), another repressive modification. Among H3K9 methyltransferases, the enzymes Suv39h1 and Suv39h2 exhibited high levels of expression at early stages of neurogenesis and their expression decreased upon differentiation. Pharmacological inhibition of these enzymes by chaetocin in AHPs reduced H3K9me3 and concomitantly decreased neuronal differentiation while increasing proliferation. Moreover, Suv39h1 and Suv39h2 knockdown in newborn cells of the adult mouse dentate gyrus by retrovirus-mediated RNA interference impaired neuronal differentiation of progenitor cells. Our results indicate that H3K9me3 and H3K9 methyltransferases Suv39h1 and Suv39h2 are critically involved in the regulation of adult hippocampal neurogenesis by controlling the differentiation of neural progenitor cells.

## Introduction

In the adult brain, the generation of new neurons has been evidenced in the hippocampus of different mammalian species including humans (Eriksson et al., 1998; Boldrini et al., 2018; Moreno-Jimenez et al., 2019; Tobin et al., 2019). In this region, radial glia-like neural stem cells (NSCs) located in the subgranular zone (SGZ) of the dentate gyrus (DG), proliferate and differentiate into granule neurons that mature and integrate into the existing circuitry and contribute to the plasticity and function of the hippocampus (Aimone et al., 2011; Toni and Schinder, 2015; Anacker and Hen, 2017; Denoth-Lippuner and Jessberger, 2021). NSCs, also known as type 1 cells, divide to generate type 2 neural progenitor cells (NPCs) that proliferate and give rise to neuroblasts that differentiate into granule neurons that distribute in the granular cell layer (GCL) of the DG, extending their dendrites into the molecular layer and their axon through the hilus to the CA3 region (van Praag et al., 2002; Kempermann et al., 2004; Zhao et al., 2008).

Adult hippocampal neurogenesis is controlled by transcriptional and epigenetic mechanisms (Hsieh, 2012; Beckervordersandforth et al., 2015; Hsieh and Zhao, 2016; Wang et al., 2016; Yao et al., 2016). Epigenetic mechanisms, which induce changes in gene expression without affecting the DNA sequences (Felsenfeld, 2014), include DNA methylation, post-translational modifications of histone tails, chromatin remodeling and regulation by non-coding RNAs (Kouzarides, 2007; Helin and Dhanak, 2013). The modifications of histone tails are dynamically regulated by sets of enzymes that act as “writers” or “erasers” to introduce or remove specific epigenetic marks, respectively, while “readers” bind to these modifications and serve as effectors. These epigenetic modifications control the level of compaction of chromatin, which can be classified as either euchromatin, corresponding to an open and transcriptionally active conformation, or heterochromatin, corresponding to a compacted and transcriptionally silent conformation (Gilbert and Allan, 2001; Allis and Jenuwein, 2016). Heterochromatin is further categorized into facultative and constitutive heterochromatin. Facultative heterochromatin (fHC) refers to regions whose compaction and silencing is dynamic during development, as occurs for genes of specific cell types and enhancers (Trojer and Reinberg, 2007; Becker et al., 2016). In contrast, constitutive heterochromatin (cHC) is established in gene-poor areas mainly at pericentromeres and telomeres, and the silencing of these regions is more universal across developmental lineages (Saksouk et al., 2015). Specific histone posttranslational modifications are enriched in heterochromatin regions, with cHC and fHC characterized by the presence of dimethylation and trimethylation of histone H3 on lysine 9 (H3K9me2 and H3K9me3) (Trojer and Reinberg, 2007; Saksouk et al., 2015; Becker et al., 2016; Wang et al., 2019). In mammals, the methylation of this residue is catalyzed by members of the family of SET domain-containing histone methyltransferases (Rea et al., 2000); Suv39h1, Suv39h2 (also called KMT1A/1B), and SETDB1 catalyze H3K9me2 and H3K9me3 (H3K9me2/me3), SETDB2 introduces H3K9me3 (Falandry et al., 2010), while G9a and GLP (also called EHMT1 and EHMT2, respectively) catalyze H3K9me1 and H3K9me2 (Tachibana et al., 2008).

H3K9me3-mediated heterochromatin has a crucial role in regulating lineage stability, but during development there is a dynamic deposition of this modification that is involved in cell type-specific regulation of fHC (Liu et al., 2015; Becker et al., 2016; Nicetto et al., 2019). High levels of compacted H3K9me3-heterochromatin are observed in uncommitted cells, and upon differentiation there is a profound rearrangement and reduction of H3K9me3 (Nicetto et al., 2019). Initially, H3K9me3-mediated heterochromatin represses cell type-inappropriate protein-coding genes (Liu et al., 2015; Nicetto and Zaret, 2019), and upon differentiation there is a progressive loss of H3K9me3 at lineage-specific genes that enables developmental cell differentiation (Nicetto et al., 2019; Nicetto and Zaret, 2019). H3K9me3 is maintained at cell type-inappropriate genes that remain silenced, thus this modification helps to maintain cellular identity (Nicetto and Zaret, 2019). Whether H3K9me3 plays a role in controlling cell differentiation in adult hippocampal neurogenesis remains unknown. Here we explored for the first time the expression of H3K9me3 during adult hippocampal neurogenesis and evaluated the role of the enzymes methyltransferases that introduce this modification in the neurogenic process. We determined that there is a dynamic deposition of H3K9 methylation during adult hippocampal neurogenesis, and that H3K9 methyltransferases Suv39h1 and Suv39h2 are critical for this process. The data also indicate that in the adult hippocampus H3K9 methylation is tightly controlled and is pivotal for cell differentiation. Together, our data contribute to the understanding of epigenetic mechanisms controlling neurogenesis in the adult hippocampus and identify possible epigenetic targets for conditions affecting this process.

## Materials and Methods

### Animals

Two-month old C57/BL6 male mice were used for all experiments. All procedures involving experimentation on animals were carried out according to NIH guidelines as well as ARRIVE guidelines and were approved by the Bioethical Committee of the Universidad Andres Bello. Mice had access to water and food ad libitum in a 12:12 h light/dark cycle.

### Isolation and Culture of Mouse Adult Hippocampal Progenitors

Adult hippocampal progenitors (AHPs) were isolated from the hippocampus of 10 female 6-week-old C57/BL6 mice and cultured in monolayers as previously described (Babu et al., 2011) with some modifications (Arredondo et al., 2020). Briefly, animals were euthanized with a mixture of ketamine/xylazine (200 mg/kg, 20 mg/kg) in saline serum, decapitated and after removing the brain and washing it in PBS, the hippocampus was dissected. Hippocampi were washed in basal medium (Neurobasal A (Gibco), supplemented with B27 without vitamin A (Gibco), Glutamax (Gibco), 100 U/ml penicillin and 100 μg/ml streptomycin and fungizone). Then the hippocampi were enzymatically digested for 35 min at 37°C using the enzymatic mixture of Papain (2.5 U/ml, Worthington), DNAse I (250 U/ml, Worthington) and Dispase I (1 U/ml, Roche) in Neurobasal A medium. The suspension was triturated with Pasteur pipettes, and then centrifuged at 1000 rpm for 5 min at room temperature. The cell suspension was resuspended in basal medium and the centrifugation was repeated. Cells were suspended in 7.8 ml of PBS with Glucose (30 mM), and 2.2 ml of a Percoll (GE Heathcare)-PBS solution (final concentration of 22% vol/vol Percoll), and centrifuged at 1500 rpm for 15 min at room temperature. The supernatant was discarded without disturbing the pellet containing the progenitor cells. Cells were washed two times in basal medium. AHP were plated at 10,000 cell/cm^2^ in culture plates pretreated with poly-D-lysine and laminin. Briefly, culture plates were incubated with 0.01 mg/ml poly-D-lysine (Gibco) overnight at room temperature. Next day, poly-D-lysine was removed and culture plates and were treated with 10 μg/ml laminin (Gibco) in DMEM/F-12 medium (Gibco) overnight at 37°C. AHP were cultured in proliferation medium consisting of DMEM/F-12 supplemented with B27 (Thermo Fisher Scientific), 100 U/ml penicillin and 100 μg/ml streptomycin and the growth factors FGF-2 (20 ng/ml, Alomone Labs) and EGF (20 ng/ml, R&D systems). Differentiation of AHPs was induced by growth factor withdrawal for 1 or 2 days. For transfection, AHPs cultured at a density of 10,000 cell/cm^2^ in 21 cm^2^ were transfected 48 h after seeding with Lipofectamine 3000 (Thermo Fisher Scientific) following manufacturer’s instructions.

### Immunofluorescence Staining

Immunodetection in cultured cells was performed as previously described (Varela-Nallar et al., 2009). For immunostaining in tissue sections, perfusion, postfixation and tissue sectioning was carried out as described in (Arredondo et al., 2020). Tissue sections were sequentially collected in 12 sets of serial slices of 40-μm thickness. Immunodetection of neurogenesis markers, epigenetic marks and BrdU was carried out as previously described (Mardones et al., 2016; Jury et al., 2020). Primary antibodies used: mouse anti-GFAP (Sigma-Aldrich, Cat. G3893, 1:8,000), goat anti-SOX2 (Santa Cruz Biotechnology, Cat. sc-365823 1:250), goat anti-DCX (Santa Cruz Biotechnology, Cat. sc-271390, 1:250), mouse anti-NeuN (Millipore, Cat. MAB377, 1:300), mouse anti-Nestin (Millipore, Cat. MAB5326, 1:100), rat anti-BrdU (Abcam, USA Cat. ab6326, 1:300), rabbit anti-ZsG (Clontech Cat. 632,474, 1:1,000), rabbit anti-H3K9me3 (Abcam, Cat. ab8898, 1:1,000), mouse anti-H3K9me2 (Abcam, Cat. ab1220, 1:1,000), rabbit anti-H3K9me1 (Abcam, Cat. ab9045, 1:1,000), rabbit anti-H3K9ac (Abcam, Cat ab4441, 1:500), rabbit anti-H3K36me3 (Abcam, Cat ab9050, 1:500), rabbit anti-active caspase 3 (BD Biosciences, Cat. 559565, 1:200). As secondary antibodies Alexa (Molecular Probes), and DyLight (Abcam) conjugated antibodies were used. NucBlue (NucB, Life Technologies, Cat. R37605) was used as nuclear dye. Slices were mounted on gelatin-coated slides with Fluoromont-G (Electron Microscopy Sciences).

### Image Acquisition and Analysis

For *in vitro* and *in vivo* analysis, images were acquired by confocal laser microscopy (Leica TCS SP8) with a 63x oil objective and a z-step of 1 μm (cultured cells) or 0.5 μm (tissue sections) optical sections. Maximum intensity projections of confocal z-stack images of whole nuclei of hippocampal cells (containing 5–7 stacks) and AHP (containing 7–10 stacks) were analyzed. The Fiji-ImageJ software (Schindelin et al., 2012) was used to analyze NucB, H3K9me3, H3K9me2, H3K9ac and H3K36me3 foci, mean nuclear area, and H3K9me1 fluorescence intensity. The quantification of foci (≥0.30 μm for cultured cells, ≥0.03 μm for tissue sections) was carried out under threshold conditions (8 bits, measuring intensity from 0 to 255; circularity 0.0–1.0) using the analyze particle plug-in of ImageJ as previously described (Jury et al., 2020). For co-distribution of H3K9me2/me3 and NucBlue, line scans were drawn in Fiji (ImageJ) software to quantify the relative intensities as previously described (Mansuroglu et al., 2016; Jury et al., 2020). In tissue sections, at least 10 to 20 isolated nuclei were analyzed for each cell type (NSCs, NPCs, neuroblasts, immature and mature neurons) in each animal. In cultured AHPs, in each experimental condition 10 or 15 microscopy fields were analyzed for the proliferative and differentiated conditions, respectively. In proliferative AHP between 60–100 nuclei were analyzed in each experiment for each experimental condition. In differentiated AHP between 40–60 nuclei of AHP-derived neurons and 100–120 nuclei of AHP-derived astrocytes were analyzed in each experiment for each experimental condition.

### Proliferation Analysis in Cultured AHPs

To analyze proliferation, AHPs were treated for 24 h with or without 1.25 and 2.5 nM chaetocin (Sigma-Aldrich) and were incubated with 10 μM BrdU (Sigma-Aldrich) for the last 2 h of treatment. Immunodetection of BrdU was carried out as previously described (Babu et al., 2011) with some modifications. The DNA was denaturated by incubating with HCl 2 M for 15 min at room temperature. Then, the cells were washed six times with PBS for 5 min at room temperature and were incubated with PBS 1% BSA for 30 min at room temperature before continuing with the immunodetection protocol. Proliferation was evaluated as the percentage of cells positive for BrdU of the total number of cells (positive for the nuclear marker NucB).

### Western Blotting

For total protein extract, AHP were lysed in RIPA buffer (10 mM Tris/HCl pH 7.4, 5 mM EDTA, 1% NP-40, 1% sodium deoxycholate and 1% SDS), supplemented with a protease (Halt protease inhibitor single-use cocktail, Thermo Scientific) and phosphatase (Halt phosphatase inhibitor cocktail, Thermo Scientific) inhibitors. Homogenates were kept on ice for 30 min and then centrifuged at 12,000 rpm for 10 min (4°C) to remove nuclei and large debris. For total nuclear protein extract, AHP were lysed in an ice-cold lysis buffer [60 mM KCl, 15 mM NaCl, 2 mM EDTA, 0.5 mM EGTA, 15 mM Tris-HCl pH 7.4, 0.5 mM spermidine (Sigma-Aldrich, Cat. S2501), 0.5 mM DTT, and 0.2% NP-40]. To obtain isolated nuclear fractions the membrane was disintegrated using the Kimble Dounce tissue grinder set (Sigma, D8938). Nuclei were centrifuged at 6000 rpm for 10 min at 4°C, and the pellet was resuspended in a sonication buffer [50 mM HEPES pH 7.9, 140 mM NaCl, 1 mM EDTA, 1% Triton X-100, 0.1% sodium deoxycholate, and 10% SDS with protease inhibitors (Roche, Cat. 11836153001)]. Sonication was performed in a Bioruptor sonicator (Diagenode) for 30 seg pulse on/off in 5 min at maximum power. Protein concentration was determined using the BCA Protein Assay Kit (Pierce, Thermo Fischer Scientific). Proteins were resolved in 12% SDS-PAGE, transferred to a PVDF membrane, blocked, and incubated overnight at 4°C with primary antibodies: rabbit anti-Suv39h1 (Cell signaling, Cat. D11B6, 1:500), rabbit anti-Suv39h2 (Abcam, Cat. ab189842, 1:500), rabbit anti-H3K9me3 (Abcam, Cat. ab8898, 1:1,000). For loading control the membrane was incubated with the ReBlot Plus Strong Antibody Stripping Solution (Millipore, United States, Cat. 2504) for 3 min. After washing three times with PBS 0.05% Tween 20, the membrane was incubated overnight at 4°C with mouse anti-β-actin (Abcam, Cat. ab8226, 1:10,000) or rabbit anti-histone H3 (Abcam, Cat. ab1791, 1:10,000). The reactions were followed by incubation with peroxidase-conjugated secondary antibodies (Pierce, Thermo Fisher Scientific) and developed using the ECL technique (Perkin Elmer, Waltham, MA, United States).

### Reverse Transcriptase and Quantitative Real-Time PCR (RT-qPCR)

Total RNA (AHP: D0, D1 and D2) was extracted using TRIzol reagent (Life Technologies) and reversely transcribed into complementary DNA (cDNA) using M-MuLV reverse transcriptase (New Englands BioLabs, Ipswich, MA, United States). qPCR was performed using Brilliant II SYBR Green QPCR master mix (Agilent Technologies, Santa Clara, CA, United States). Primers used were: Suv39h1: 5′-CCT​GCC​CTT​GGT​GTT​TCT​AA-3′ (forward), 5′-CAC​GCC​ACT​TAA​CCA​GGT​AAT​A-3′ (reverse); Suv39h2: 5′-GCT​GGA​GAA​GAG​CTG​ACT​TT-3′ (forward), 5′- AAG​TCT​CGG​CTC​CAC​ATT​TAC-3′ (reverse); G9a: 5′- ACA​ACG​CAC​GCC​ACT​AAT-3′ (forward), 5′- CAT​CCT​CTT​CCT​TGC​TGT​AGA​C-3′ (reverse); SETDB1: 5′- TAT​CCG​CTG​CTT​GGA​TGA​TAT​T-3′ (forward), 5′- CAT​CTC​CAG​GCC​TTC​TTT​GT-3′ (reverse); SETDB2: 5′- CTG​AGG​GCT​GCA​TAG​ACA​TAA​A-3′ (forward), 5′- ATA​TCC​AGC​ACA​TTC​TCC​ATC​C-3′ (reverse); NeuroD1: 5′-CCT​GAT​CTG​GTC​TCC​TTC​GTA-3′ (forward), 5′-CAA​GAA​AGT​CCG​AGG​GTT​GA-3′ (reverse); GAPDH: 5′-CAT​GGC​CTT​CCG​TGT​TCC​TA-3′ (forward), 5′-CCT​GCT​TCA​CCA​CCT​TCT​TGA​T-3′ (reverse); actin: 5′-AAGGCCAACCGTGAAAAGAT-3′(forward), 5′-GTG​GTA​CGA​CCA​GAG​GCA​TAC-3′ (reverse). mRNA levels were estimated using the 2^−ΔΔCT^ method and normalized to the GAPDH gene or to the mean expression of GAPDH and actin genes (Rao et al., 2013).

### Retrovirus Production and Stereotaxic Surgery

To knockdown Suv39h1 and Suv39h2, an inverted and self-complementary hairpin DNA oligonucleotide encoding a short-hairpin RNA targeting mouse Suv39h1 and mouse Suv39h2 mRNAs were chemically synthesized and cloned by Genscript into the retroviral vector pSIREN-RetroQ (Clontech) that coexpress the fluorescent protein ZsGreen (ZsG). As a control, an shRNA targeting Luciferase mRNA (sh-Ctrl) provided by the manufacturer (Clontech) was used. The retrovirus used lack nuclear import mechanisms, thus viral integration occurs only in proliferating cells (Lewis and Emerman, 1994). Oligos used to construct the shRNA were: sh1-Suv39h1: 5′-GAT​CCG*CTG​CAC​AAG​TTT​GCC​TAC​AAT​GA*TTC​AAG​AGA​TCA​TTG​TAG​GCA​AAC​TTG​TGC​AGC​TTT​TTT​ACG​CGT​G-3′; sh2-Suv39h1: 5′-GAT​CCG*GAC​TAC​GTG​GAA​GAC​GTA​TAT​AC*TTC​AAG​AGA​GTA​TAT​ACG​TCT​TCC​ACG​TAG​TCC​TTT​TTT​ACG​CGT​G-3′; sh1-Suv39h2: forward: 5′-GAT​CCG*TGC​AGC​TCG​ATA​TGG​AAA​CGT​AT*TTC​AAG​AGA​ATA​CGT​TTC​CAT​ATC​GAG​CTG​CAC​TTT​TTT​ACG​CGT-G-3′; sh2-Suv39h2: forward: 5′-GAT​CCG*CGG​GGC​ACA​TAA​ACG​GTA​GAT​AT*TTC​AAG​AGA​ATA​TCT​ACC​GTT​TAT​GTG​CCC​CGC​TTT​TTT​ACG​CGT​G-3′. Each target mRNA sequence is shown in italic. Retroviral particles were prepared as previously described (Tashiro et al., 2006) with some modifications (Mardones et al., 2019). Retroviral pellets were resuspended in PBS and aliquots were immediately stored at −80°C.

Before surgery, 2-month-old mice were deep anesthetized using a mixture of ketamine/xylazine (150 mg/kg, 15 mg/kg) and placed in a stereotaxic frame (RWD, Life Science Co.). Retroviruses (1.5 μl) were injected unilaterally into the dorsal and ventral DG using the following coordinates (Jang et al., 2013): dorsal: ±1.5 mm lateral, −2 mm anterioposterior, −2.3 mm depth; ventral: ±2.6 mm lateral; −3 mm anterioposterior; −3.2 mm depth for caudal DG from bregma.

### Differentiation Analysis *In Vitro* and *In Vivo*

To analyze neuronal differentiation of newborn cells *in vivo*, animals were sacrificed 1 week post injection (wpi). Brain sections were processed for immunostaining and analyzed by confocal laser microscopy (Leica TCS SP8) with a 63x oil objective and a z-step of 1 μm optical sections. The expression of protein markers DCX, GFAP and Sox2 was analyzed in ZsG-positive cells (5–7 z-stacks) using the ImageJ software. For quantification, all ZsG-positive cells in one set of tissue sections were analyzed, and the percentage of ZsG-positive cells of each cell-type was estimated. For that, the expression of protein markers and the morphology of cells were used to identify NSCs (GFAP+/Sox2+ radial morphology), NPCs (GFAP-/Sox2+ spheric morphology in SGZ), neuroblasts (DCX+ horizontal projections in SGZ) and immature neurons (DCX+ dendritic projections through GCL). For the *in vitro* analysis, cultured AHP were differentiated by removing the growth factors (FGF-2 and EGF) from the medium. The percentage of cells positive for DCX or GFAP staining of the total number of cells (positive for the nuclear marker NucB) was quantified.

### Statistical Analysis

Statistical analyses were performed using Prism8 software (GraphPad Software Inc.). Data normality was checked using the Shapiro-Wilk test; all data showed normal distribution. One-way ANOVA followed by Bonferroni multiple comparison posthoc test, was used for comparison between more than two groups. *p* < 0.05 was considered statistically significant. In all graphs the data represent the mean ± SEM. Number of independent experimental replicates, the test used, and the *p* values are indicated in each figure legend.

## Results

### Changes in the Expression and Distribution of H3K9me3 Throughout the Stages of Adult Hippocampal Neurogenesis

To study the distribution of the repressive epigenetic modification H3K9me3 during the stages of the neurogenic process in the DG of 2-month-old mice, we carried out H3K9me3 immunofluorescence staining (Calderon-Garciduenas et al., 2020; Jury et al., 2020), with cells at the different stages of neurogenesis identified by specific protein markers. The nuclear staining NucBlue (NucB) was used to evaluate the global chromatin organization, particularly of the pericentric heterochromatin clusters or chromocenters, formed by the aggregation of heterochromatin from multiple chromosomes. The presence of H3K9me3 is a conserved hallmark within chromocenters, giving rise to H3K9me3-positive foci (Peters et al., 2003; Saksouk et al., 2015; Mansuroglu et al., 2016; Jury et al., 2020).

**FIGURE 1:**
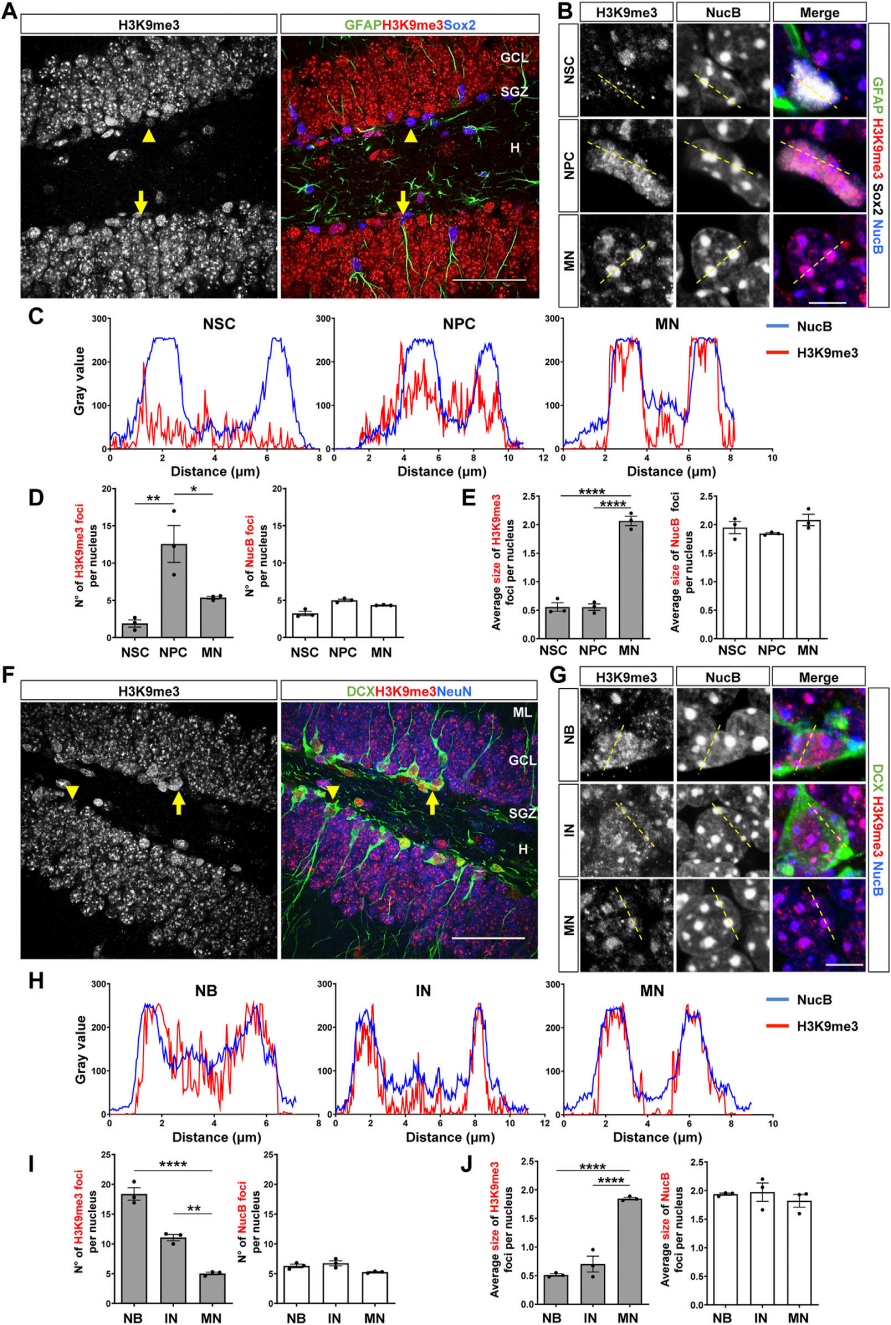
Nuclear H3K9me3 staining and distribution during the stages of neurogenesis in the adult mouse hippocampus. **(A)** Immunostaining of H3K9me3, GFAP and Sox2 in the DG of 2-month-old mouse. The arrow indicates a NSC (GFAP+Sox2+), the arrowhead indicates an NPC (GFAP-Sox2+). Scale bar 50 µm. Higher magnifications are shown in Supplementary Figure S1A. **(B)** Digital zoom of nuclei from NSC (top), NPC (middle) and mature neuron (MN, bottom). GFAP (green), H3K9me3 (red), Sox2 (white) NucB (blue). Dotted lines indicate the line scans analyzed in C. Scale bar 10 µm. **(C)** Fluorescence intensity of H3K9me3 (red) and NucB (blue) in line scans drawn across chromocenters in nuclei from NSC, NPC and MN using a single confocal section. **(D)** Average number of H3K9me3 and NucB foci per nucleus of NSC, NPC and MN. **(E)** Average size of H3K9me3 and NucB foci in the different cell types. **(F)** Immunofluorescence staining of H3K9me3, DCX and NeuN in the DG. Arrow indicates a neuroblast and the arrowhead indicates an immature neuron. Scale bar 50 µm. Higher magnifications are shown in Supplementary Figure S1B. **(G)** Digital zoom of nuclei from neuroblast (top), immature neuron (middle) and MN (bottom). DCX (green), H3K9me3 (red), NucB (blue). Dotted lines indicate the line scans analyzed in H. Scale bar 10 µm. **(H)** Fluorescence intensity of H3K9me3 (red) and NucB (blue) in line scans drawn across chromocenters in nuclei from neuroblasts (NB), immature neurons (IN) and MN using a single confocal section. **(I)** Average number of H3K9me3 and NucB foci per nucleus of NB, IN and MN. **(J)** Average size of H3K9me3 and NucB foci in the different cell types. Bars show mean ± SEM. Dots show individual data. *N* = 3 mice. *p < 0.05; **p < 0.01, ****p < 0.0001, one-way ANOVA followed by Bonferroni posthoc test. ML: Molecular layer; GCL: Granule cell layer; SGZ: Subgranular zone, H: Hilus.

NSCs and NPCs were identified by immunostaining with GFAP and Sox2 and morphology (Seri et al., 2001; Zhao et al., 2008). NSCs are positive for GFAP and Sox2 and extend a radial process into the GCL (Figure 1A, arrow, Supplementary Figure S1A), while NPCs are rounded cells positive for Sox2 but not for GFAP, and are located in the SGZ (Figure 1A, arrowhead, Supplementary Figure S1A). H3K9me3 staining in these cells was compared with that in mature neurons (MN) of the outer third of the GCL, where adult-born neurons are not incorporated (Esposito et al., 2005; Kempermann et al., 2003). H3K9me3 showed a diffuse staining in NSCs nuclei (Figure 1B, top panels), while a more clustered distribution of H3K9me3 was observed in the nucleus of NPCs (Figure 1B, middle panels). In mature neurons, intense foci of H3K9me3 staining were observed (Figure 1B, bottom panels). To analyze co-distribution of H3K9me3 staining and NucB, line scans were drawn across the chromocenters to quantify the relative intensities of the fluorescence signals (Mansuroglu et al., 2016; Jury et al., 2020). In mature neurons, H3K9me3 foci were co-distributed with NucB-intense foci corresponding to pericentromeric heterochromatin (Figure 1C), while no or little co-distribution was observed in NSCs and NPCs, respectively (Figure 1C). The number and size of H3K9me3 and NucB foci per nucleus were analyzed (Figures 1D,E). A significant increase in the number of H3K9me3 foci was observed in NPCs compared to NSCs, which was reduced in mature neurons (Figure 1D). However, mature neurons exhibited larger size of H3K9me3 foci compared to NSCs and NPCs (Figure 1E). No changes in the number or size of NucB foci were observed between the different cell types (Figures 1D,E), suggesting that changes in H3K9me3 distribution are not accompanied by robust changes in cHC.

The same analysis was carried out in neuroblast and immature neurons, which were identified by immunostaining with DCX and NeuN, and cell morphology (Encinas et al., 2011; Zhao et al., 2008). Neuroblast are rounded cells positive for DCX but negative for NeuN, are located in the SGZ and may exhibit small horizontal processes (Brown et al., 2003) (Figure 1F, arrow, Supplementary Figure S1B). Immature neurons are positive for DCX, may or may not express NeuN based on their maturation, and are in the GCL extending dendrites across the GCL and molecular layer (Brown et al., 2003; Kempermann et al., 2004) (Figure 1F, arrowhead, Supplementary Figure S1B). A clustered distribution of H3K9me3 was observed in both cell types (Figure 1G, top and middle panels); for comparison, H3K9me3 in mature neurons is also shown (Figure 1G, bottom panels). Line scans showed co-distribution of H3K9me3 and NucB foci in neuroblasts, which was more evident in immature and mature neurons (Figure 1H). Compared to mature neurons, neuroblast and immature neurons showed a significantly higher number of H3K9me3 foci (Figure 1I), however the average size of H3K9me3 foci was significantly larger in mature neurons (Figure 1J). No changes in the number or size of NucB foci were observed among cell types (Figures 1I,J), suggesting there are no robust changes in cHC. Altogether, these results indicate that the distribution of H3K9me3 changes in the different stages of adult hippocampal neurogenesis.

**FIGURE 2:**
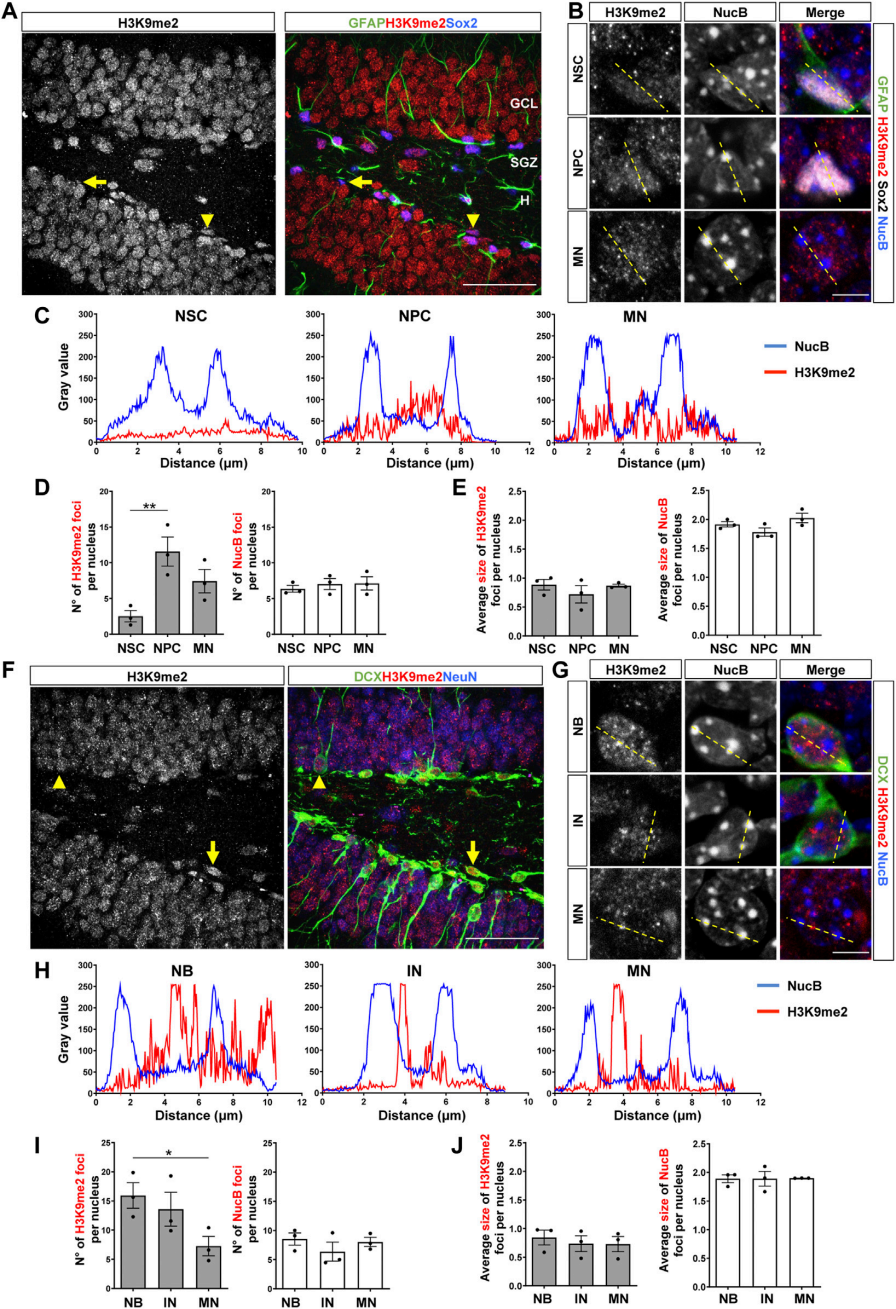
Nuclear H3K9me2 staining and distribution during the stages of neurogenesis in the adult mouse hippocampus. **(A)** Immunostaining of H3K9me2, GFAP and Sox2 in the DG of 2-month-old mouse. The arrow indicates a NSC (GFAP+Sox2+), the arrowhead indicates a NPC (GFAP-Sox2+). Scale bar 50 µm. Higher magnifications are shown in Supplementary Figure S2A. **(B)** Digital zoom of nuclei from NSC (top), NPC (middle) and mature neuron (MN, bottom). GFAP (green), H3K9me2 (red), Sox2 (white) NucB (blue). Dotted lines indicate the line scans analyzed in C. Scale bar 10 µm. **(C)** Fluorescence intensity of H3K9me2 (red) and NucB (blue) in line scans drawn across chromocenters in nuclei from NSC, NPC and MN using a single confocal section. **(D)** Average number of H3K9me2 and NucB foci per nucleus of NSCs, NPCs and MN. **(E)** Average size of H3K9me2 and NucB foci in the different cell types. **(F)** Immunofluorescence staining of H3K9me2, DCX and NeuN in the DG. Arrow indicates a neuroblast and the arrowhead indicates an immature neuron. Scale bar 50 µm. Higher magnifications are shown in Supplementary Figure S2B. **(G)** Digital zoom of nuclei from neuroblast (top), immature neuron (middle) and MN (bottom). DCX (green), H3K9me2 (red), NucB (blue). Dotted lines indicate the line scans analyzed in H. Scale bar 10 µm. **(H)** Fluorescence intensity of H3K9me2 (red) and NucB (blue) in line scans drawn across chromocenters in nuclei from neuroblasts (NB), immature neurons (IN) and MN using a single confocal section. **(I)** Average number of H3K9me2 and NucB foci per nucleus of NB, IN and MN. **(J)** Average size of H3K9me2 and NucB foci in the different cell types. Data are presented as mean ± SEM; *N* = 3 mice. *p < 0.05; **p < 0.01, one-way ANOVA followed by Bonferroni posthoc test. ML: Molecular layer; GCL: Granule cell layer; SGZ: Subgranular zone, H: Hilus.

The distribution of H3K9me2 was also evaluated in the adult DG. In previous studies, compared to the H3K9me3 staining, the immunofluorescence signal for the H3K9me2 modification in adult neurons was detected in smaller and diffuse foci that do not co-localize with cHC (Poleshko et al., 2019; Jury et al., 2020). Here, we also found in NSCs (Figure 2A, arrows, Supplementary Figure S2A) that H3K9me2 showed a diffuse staining (Figure 2B, top panels), while in NPCs (Figure 2A, arrowhead, Supplementary Figure S2A) and mature neurons some intense, but small, H3K9me2 foci were observed (Figure 2B). No co-distribution of H3K9me2 and NucB foci were observed in NSCs, NPCs or mature neurons (Figure 2C). The number of H3K9me2 foci per nucleus were significantly higher in the NPCs compared to the NSCs and decreased in mature neurons (Figure 2D). No changes in the size of H3K9me2 foci were observed between the different cell types (Figure 2E). In neuroblasts (Figure 2F, arrow, Supplementary Figure S2B) and immature neurons (Figure 2F, arrowhead, Supplementary Figure S2B) H3K9me2 showed a diffuse staining (Figure 2G), and no co-distribution of H3K9me2 and NucB foci was observed (Figure 2H). Neuroblasts showed higher number of H3K9me2 foci compare to mature neurons (Figure 2I), but no changes in the size of H3K9me2 foci were observed among the different cell types (Figure 2J). Again, no changes in the number or size of NucB foci were observed between the cell types analyzed (Figures 2D,E,I,J).

**FIGURE 3:**
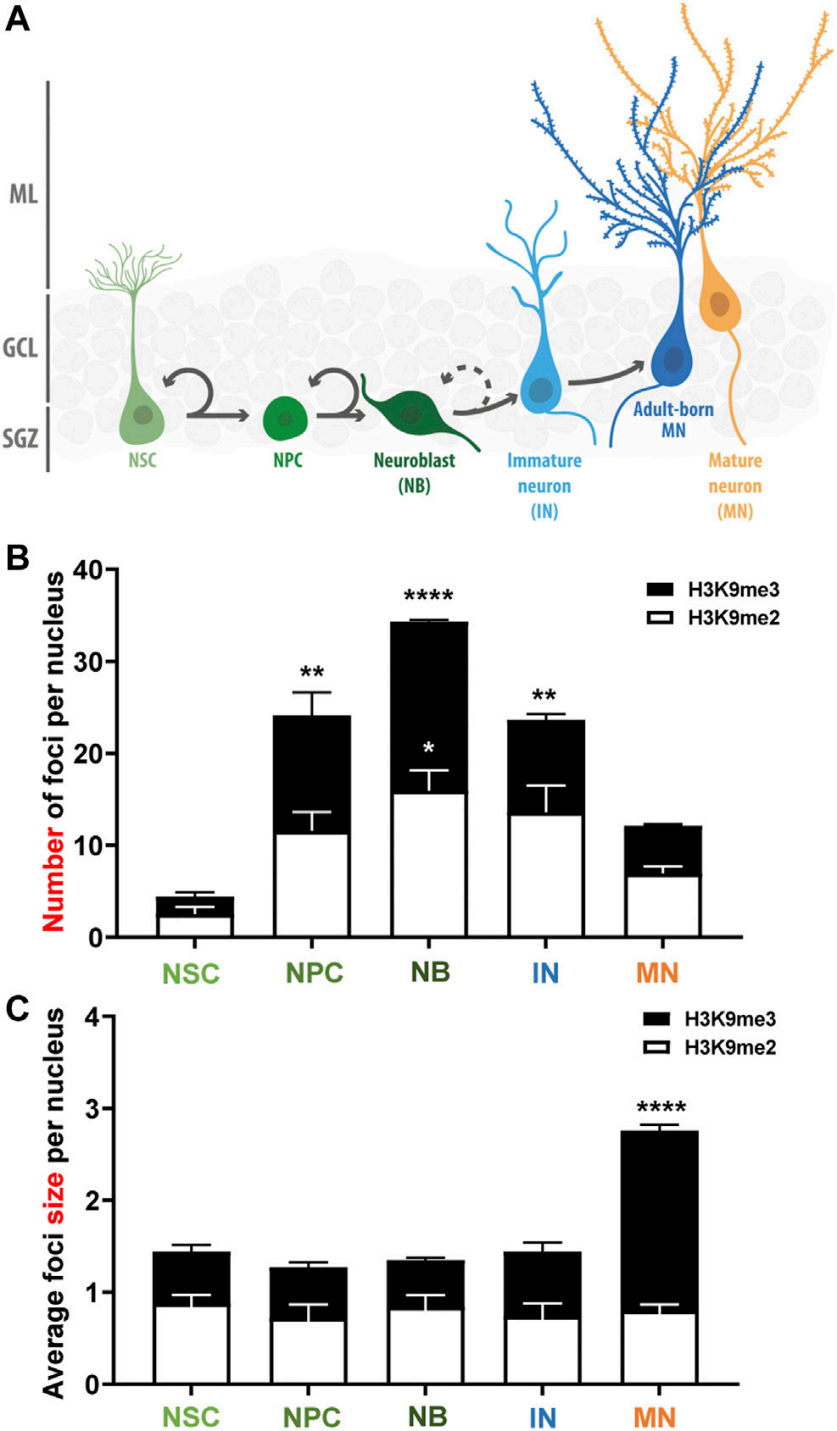
Changes in the number and size of H3K9me2/me3 foci during the stages of adult neurogenesis in the mouse hippocampus. **(A)** Schematic representation of the adult neurogenesis process in the adult mouse dentate gyrus. Neural stem cells (NSC) proliferate asymmetrically to generate neural progenitor cells (NPC) that give rise to neuroblasts (NB) that differentiate morphologically into immature granule neurons (IN) that became mature neurons (adult born MN). In our analyses, H3K9me2/me3 staining was analyzed in NSC, NPC, NB and IN, and in mature neurons of the outer third of the GCL (MN) which are MN generated during embryonic development. **(B,C)** Average number of H3K9me2 (white bars) and H3K9me3 (black bars) foci per nucleus **(B)** and foci size **(C)** in the different cell-types during the progression of neurogenesis. Bars show mean ± SEM. Statistical differences compared to the NSCs are indicated. *p < 0.05; **p < 0.01, ****p < 0.0001, one-way ANOVA followed by Bonferroni posthoc test, *N* = 3 mice. SGZ: Subgranular zone; GCL: Granule cell layer; ML: Molecular layer.

These results suggest that there is a dynamic deposition of H3K9me2/me3 during the stages of neurogenesis (Figure 3). A high number of small HeK9me2/3 foci is observed during fate commitment (NPCs and neuroblasts), and after differentiation there is a reduction in the number of foci concomitantly with an increase in foci size, suggesting a redistribution of these repressive histone tail marks. Moreover, we evaluated H3K9me1 during stages of neurogenesis, and although this modification shows a diffuse pattern of staining with no foci (Supplementary Figure S3), the intensity of H3K9me1 staining was higher in NPCs compared to NSCs, and in neuroblasts compared to mature neurons. Altogether, the data suggest that there is a rearrangement of H3K9 methylated epigenome during the neurogenic process in the adult hippocampus.

On the other hand, we evaluate the classical active epigenetic marks H3K36me3 and H3K9ac. H3K36me3 is enriched across the body of active genes (Carrozza et al., 2005; Joshi and Struhl, 2005), while H3K9ac is associated to active promoters and enhancers (Karmodiya et al., 2012). The number of H3K36me3 foci was significantly higher in mature neurons compared to NSCs, NPCs, neuroblast and immature neurons (Supplementary Figure S4), suggesting that deposition of H3K36me3 would contribute to the epigenetic mechanisms controlling the increased transcriptional activity in mature neurons. No changes were observed in the number or size of H3K9ac foci in the different stages of adult hippocampal neurogenesis (Supplementary Figure S5).

Altogether, the *in situ* analysis of histone posttranslational modifications revealed specific patterns of expression and nuclear distribution during the stages of adult hippocampal neurogenesis, suggesting a dynamic deposition and redistribution of these modifications during the neurogenic process.

### Reduced Expression of H3K9me3 and H3K9 Methyltransferases After Differentiation of Cultured Adult Hippocampal Progenitors

**FIGURE 4:**
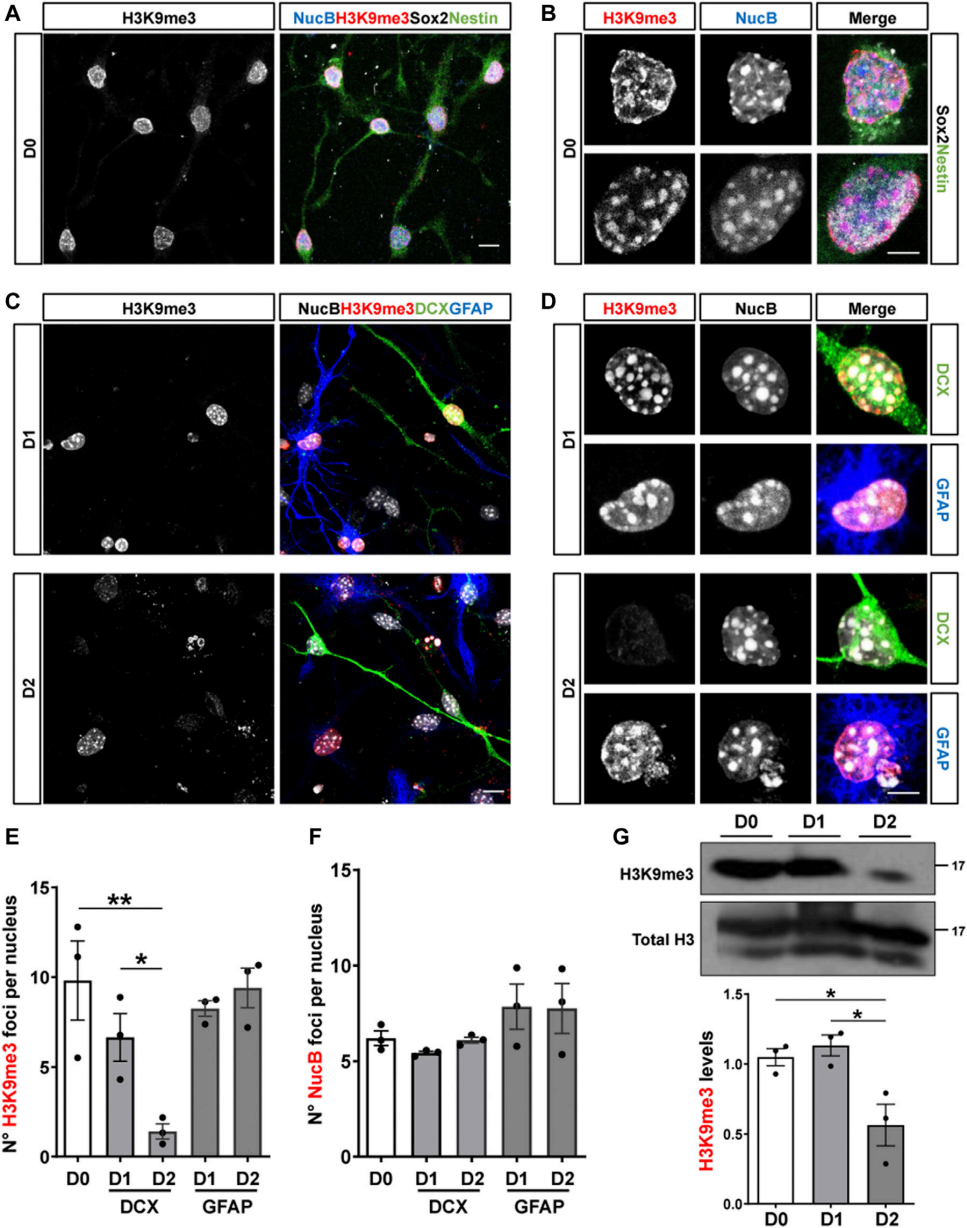
Reduced expression of H3K9me3 upon differentiation of cultured adult hippocampal progenitors. **(A,B)** Immunofluorescence staining of H3K9me3 (red), Sox2 (white), Nestin (green) and the nuclear staining NucB (blue) in proliferative (undifferentiated, D0) AHP. Scale bar: 10 μm **(A)**, 5 μm **(B)**. **(C,D)** Immunostaining of H3K9me3 (red), DCX (green), GFAP (blue) and NucB (white) in AHP differentiated by growth factor withdrawal for 24 (D1) and 48 (D2) h. Scale bar: 10 μm **(C)**, 5 μm **(D)**. **(E,F)** Average number of H3K9me3 **(E)** and NucB **(F)** foci per nucleus in AHP at D0, D1 and D2. **(G)** Immunoblot analysis of H3K9me3 in nuclear extracts from AHP at D0, D1 and D2. H3K9me3 protein levels were normalized to total H3. Numbers on the right indicate molecular weight (kDa). Data are presented as mean ± SEM; *N* = 3 independent experiments. *p < 0.05, **p < 0.01, one-way ANOVA followed by Bonferroni posthoc test.

To get insights into the mechanisms involved in the regulation of H3K9me3 in adult hippocampal neurogenesis, we carried out *in vitro* studies using adult hippocampal progenitors (AHPs) isolated from the hippocampus of adult mouse (Babu et al., 2011), that are maintained in the presence of 20 ng/ml EGF and 20 ng/ml FGF-2 and are induced to differentiate by growth factors withdrawal (Arredondo et al., 2020). H3K9me3 staining was evaluated in proliferative AHPs (D0, Figures 4A,B), positive for the cytoskeletal protein nestin and Sox2, and after 24 (D1) and 48 (D2) h of growth factor withdrawal in cells differentiated into neurons or astrocytes identified by the expression of DCX and GFAP, respectively (Figures 4C,D). A significant decrease in the number of H3K9me3 foci was observed in AHP-derived neurons at D2, which was not observed in cells differentiated into astrocytes (Figures 4C–E). No changes were observed in the number of NucB foci (Figure 4F), suggesting no global changes in cHC upon differentiation. Levels of H3K9me3 were also analyzed by immunoblot in total nuclear extracts (Figure 4G). Densitometric analysis revealed a significant decrease in H3K9me3 levels at D2 (Figure 4G), indicating that a reduction in the nuclear H3K9me3 staining signal is the consequence of a decrease in the total amount of this repressive mark, and not a redistribution.

**FIGURE 5:**
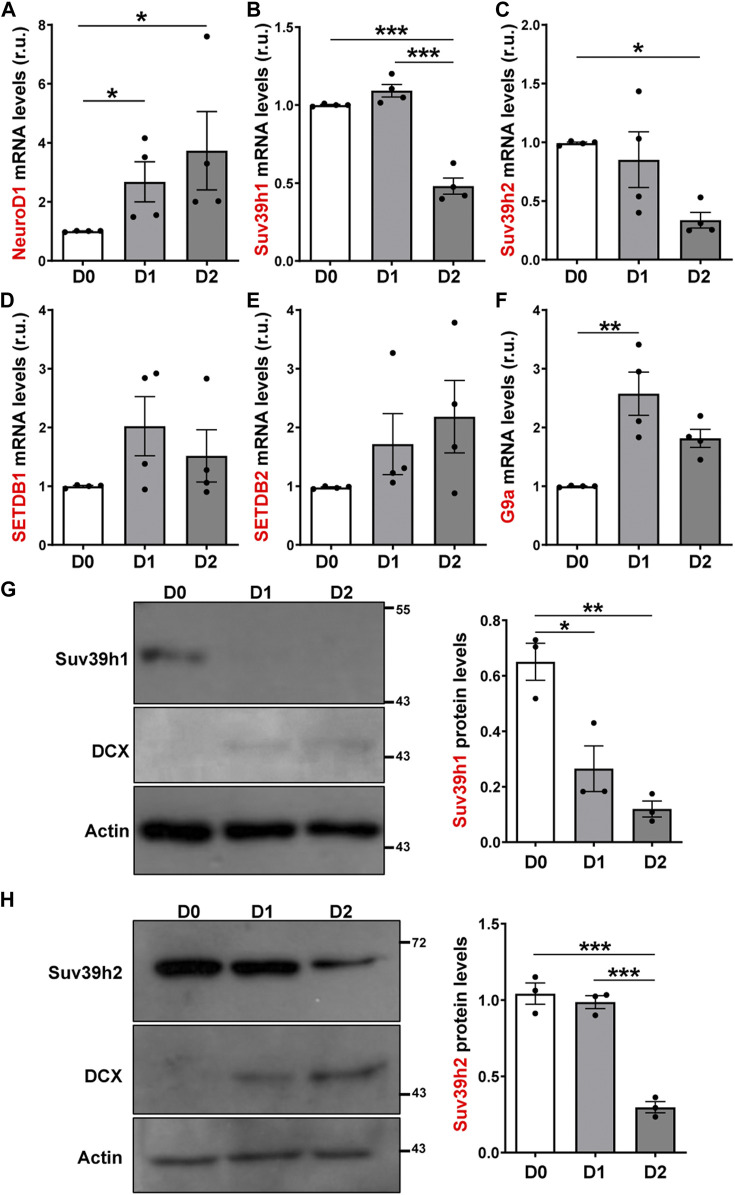
Expression of H3K9 methyltransferases in cultured adult hippocampal progenitors. **(A–F)** RT-qPCR from total RNA isolated from AHP undifferentiated (D0) or differentiated by growth factor withdrawal for 24 (D1) and 48 (D2) h. mRNA levels of NeuroD1 **(A)**, Suv39h1 **(B)**, Suv39h2 **(C)**, SETDB1 **(D)** y SETDB2 **(E)** and G9a **(F)**, were normalized to GAPDH mRNA and expressed relative to D0. **(G, H)** Immunoblot analysis of Suv39h1 **(G)** and Suv39h2 **(H)** in total protein extracts from AHP at D0, D1 and D2. DCX was used as a positive control of differentiation. Numbers on the right indicate molecular weight (kDa). Suv39h1/h2 protein levels were normalized to actin. Data are presented as mean ± SEM. *N* = 4 (RT-qPCR) or *N* = 3 (immunoblot) independent experiments. *p < 0.05, **p < 0.01, ***p < 0.001, one-way ANOVA followed by Bonferroni posthoc test.

Considering the changes in H3K9me3 levels in AHPs after differentiation, we analyzed the expression of the enzymes that write this modification (Black et al., 2012; Rao et al., 2017) by RT-qPCR. The expression of the pro-neural transcription factor NeuroD1 was used as a positive control of differentiation (Figure 5A). The mRNA levels of Suv39h1 and Suv39h2 (Suv39h1/h2), that deposit H3K9me2/me3 were significantly decreased at D2 of differentiation (Figures 5B,C). The mRNA levels of SETDB1, that write H3K9me1/me2/me3, and SETDB2, that introduce H3K9me3, showed no significant differences upon differentiation (Figures 5D,E). Also, we evaluated mRNA levels of G9a that introduce H3K9me1/me2 and observed a transient increase at D1 of differentiation (Figure 5F). Based on the RT-qPCR results, the expression of Suv39h1/h2 was further evaluated by immunoblot (Figures 5G,H). DCX protein levels was used as a positive control of differentiation. A significant decrease in Suv39h1 (Figure 5G) and Suv39h2 (Figure 5H) protein levels was observed upon differentiation of AHPs. Altogether, these results suggest that reduction of H3K9me3 is required for neurogenesis.

### Pharmacological Inhibition of Suv39h1/h2 Reduces Differentiation and Induces Proliferation of Cultured Adult Hippocampal Progenitors

**FIGURE 6:**
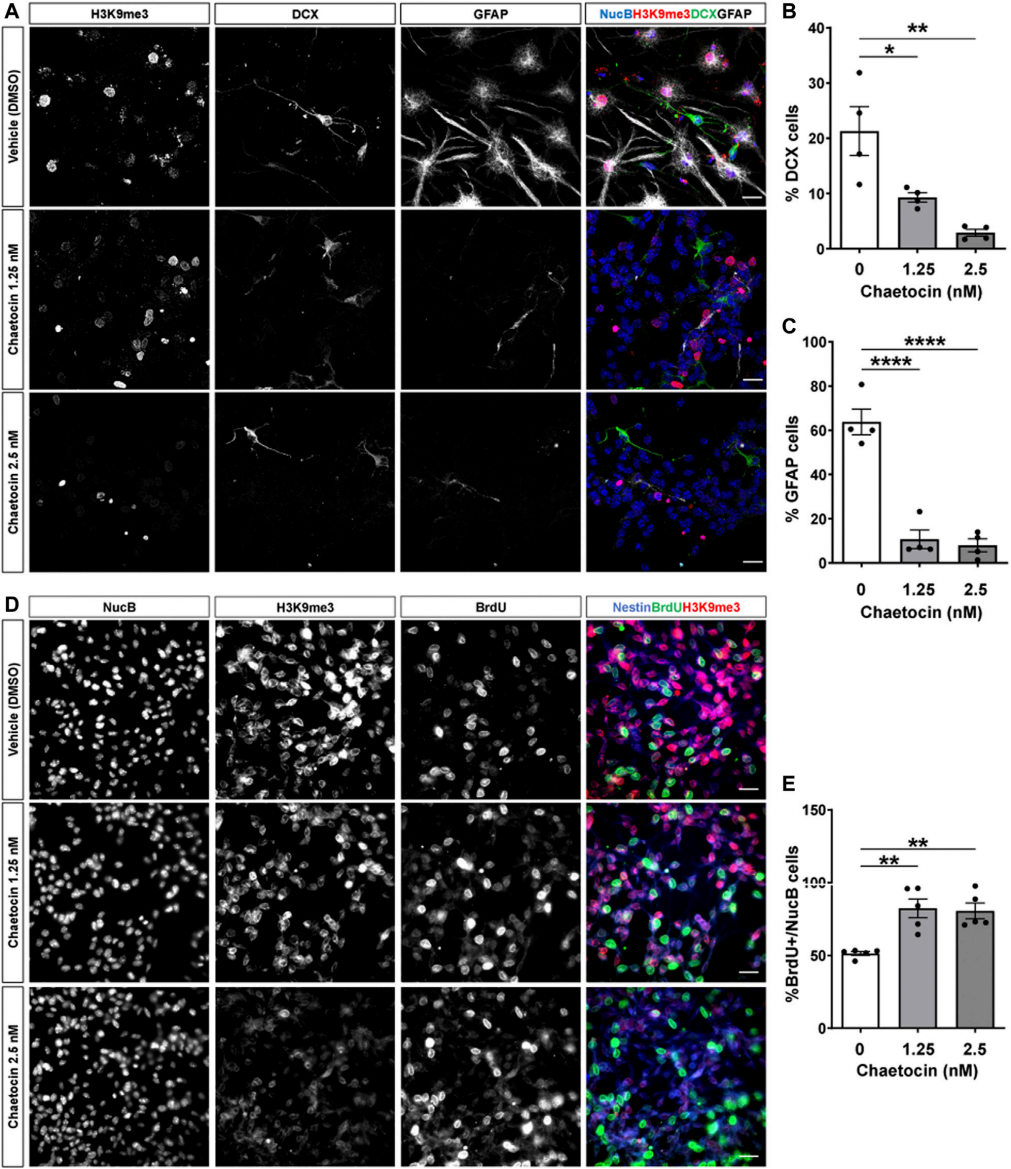
Chaetocin induces proliferation and reduces differentiation of cultured adult hippocampal progenitors. **(A)** Immunostaining of H3K9me3 (red), DCX (green) and GFAP (white) and NucB (blue) in AHP differentiated for 24 h in the presence or absence of 1.25 and 2.5 nM chaetocin. Scale bar: 20 μm. **(B,C)** Quantification of the percentage of NucB-positive cells positive for DCX (b) or GFAP (c). **(D)** AHP were treated for 24 h with or without 1.25 and 2.5 nM chaetocin and were incubated with 10 μM BrdU for the last 2 h of treatment. Images show immunostaining of H3K9me3, BrdU and the nuclear staining NucB. Merged images show H3K9me3 (red), BrdU (green) and the progenitor cell marker nestin (blue). Scale bar: 20 μm. **(E)** Quantification of the percentage of NucB-positive cells that incorporated BrdU. Data are presented as mean ± SEM; *N* = 4 (b, c) or *N* = 5 (e) independent experiments. *p < 0.05, **p < 0.01, ****p < 0.0001, one-way ANOVA followed by Bonferroni posthoc test.

To evaluate the potential role of Suv39h1/h2 in neurogenesis, we first used a pharmacological approach. Different small molecules have been developed for clinical epigenetic therapies (Helin and Dhanak, 2013). Chaetocin is an inhibitor of Suv39h1/h2 (Tran et al., 2013; Lai et al., 2015), but at higher concentration (2.5 μM) might also inhibit G9a activity (Greiner et al., 2005). To evaluate the effect of chaetocin in differentiation of AHPs, cells were induced to differentiate for 24 h in the presence or absence of 1.25 and 2.5 nM chaetocin (Figure 6A). A significant decrease in the percentage of cells positive for DCX (Figure 6B) and GFAP (Figure 6C) was observed in AHPs treated with the drug, strongly supporting that Suv39h1/h2 activity is required for AHPs differentiation. As expected, a significant decrease in the number of H3K9me3 foci were observed in cells differentiated into neurons and astrocytes, while no effect was observed in the number of NucB foci (Supplementary Figure S6). Also, we evaluated the effect of chaetocin in proliferation. AHPs were treated for 24 h with or without 1.25 and 2.5 nM chaetocin and were incubated with the nucleotide analog BrdU (10 μM) for the last 2 h of treatment. Concomitantly with a decrease in global H3K9me3 staining (Figure 6D), treatment with chaetocin significantly increased the percentage of cells positive for BrdU (Figure 6E), indicating an induction of cell proliferation. Altogether, these results indicate that the inhibitor of Suv39h1/h2 chaetocin induced a decrease in the differentiation of AHPs while inducing their proliferation capacity.

### Suv39h1 and Suv39h2 Knockdown Reduces Differentiation of Neural Precursor Cells in the Adult Mouse Dentate Gyrus

To evaluate the role of Suv39h1/h2 in adult hippocampal neurogenesis, the expression of these enzymes was knocked down in neural stem/progenitor cells using retroviral-mediated RNA interference. First, knockdown efficiency was evaluated *in vitro* in AHPs transfected with the retroviral vectors co-expressing the fluorescent protein ZsGreen (ZsG) and a control shRNA (shCtrl) or shRNAs targeting Suv39h1 (sh1-Suv39h1, sh2-Suv39h1) and Suv39h2 (sh1-Suv39h2, sh2-Suv39h2). After 48 h the expression of the enzymes was evaluated by RT-qPCR (Supplementary Figure S7). A significant reduction in Suv39h1 mRNA levels was observed in AHPs transfected with sh1-Suv39h1 and sh2-Suv39h1 (Supplementary Figure S7A), while only sh2-Suv39h2 reduced the expression of Suv39h2 (Supplementary Figure S7B). Based on these results, sh1-Suv39h1 and sh2-Suv39h2 were selected for further analyses. A decrease in the number of H3K9me3 foci was observed in the nuclei of AHPs expressing sh1-Suv39h1 and sh2-Suv39h2 with no changes in the number of NucB foci (Supplementary Figures S7C–E), supporting that Suv39h1 and Suv39h2 are critical in depositing the H3K9me3 modification in AHPs.

**FIGURE 7:**
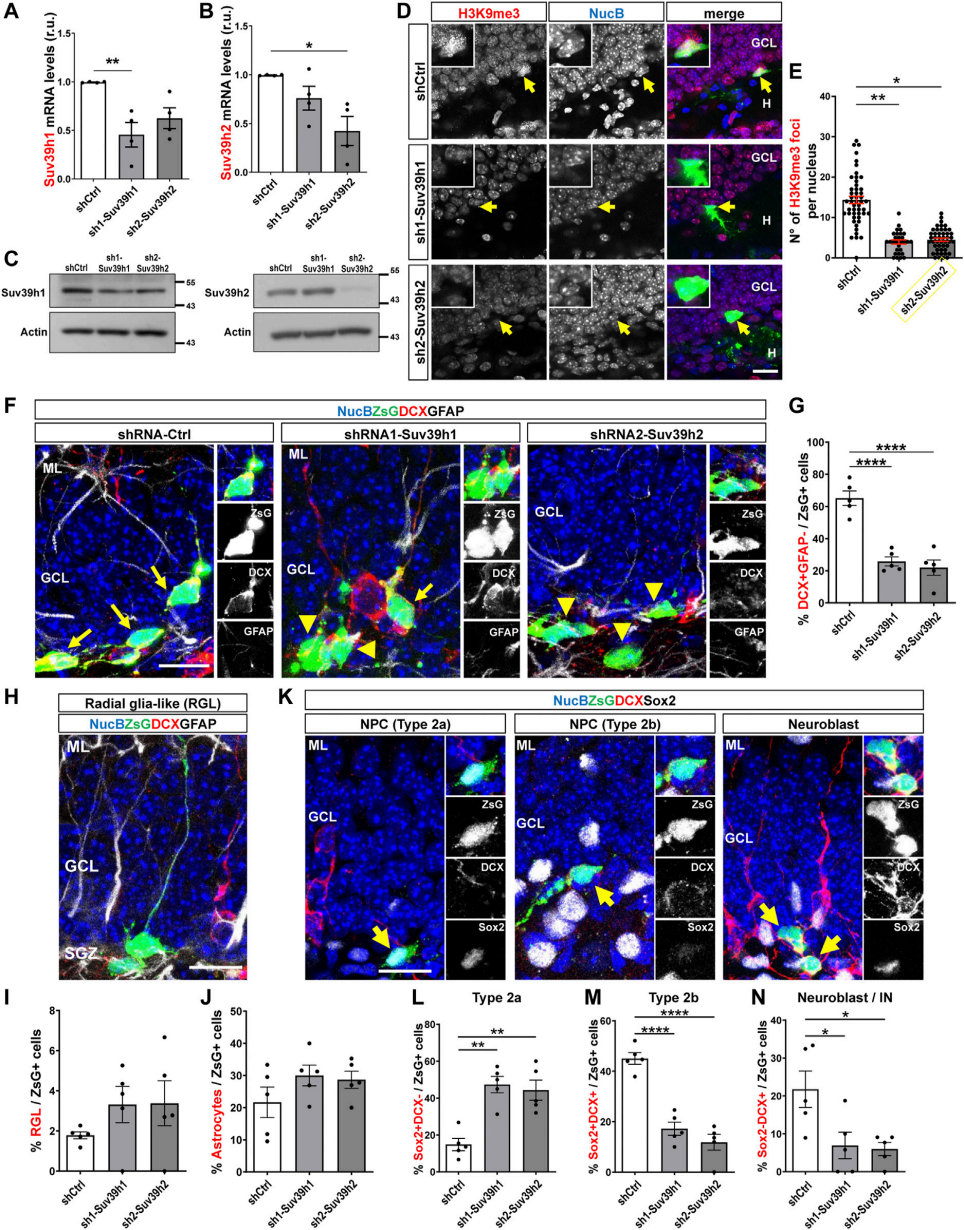
Suv39h1 and Suv39h2 knockdown reduces differentiation of NPC in the adult mouse hippocampus. **(A,B)** RT-qPCR from total RNA isolated from AHP 48 h after being transduced with retroviruses expressing shCtrl, sh1-Suv39h1 or sh2-Suv39h2. mRNA levels of Suv39h1 **(A)** or Suv39h2 **(B)** were normalized to the mean value of GAPDH and actin, and expressed relative to AHP transduced with shCtr-expressing retrovirus. Data are presented as mean ± SEM; *N* = 4 independent experiments. **(C)** Immunoblot analysis of Suv39h1 and Suv39h2 in total protein extracts from AHP transduced for 48 h with retroviruses expressing shCtrl, sh1-Suv39h1 or sh2-Suv39h2. Actin was used as loading control. Numbers on the right indicate molecular weight (kDa). **(D)** Immunostaining of H3K9me3 (red) and ZsG (green) in the DG 1 week post injection of retroviruses expressing shCtr, sh1-Suv39h1 or sh2-Suv39h2. NucB (blue) was used as nuclear staining. Scale bar: 20 μm. Insets show higher magnifications of infected cells (positive for ZsG, arrow). **(E)** Average number of H3K9me3 foci per nucleus. Data are presented as mean ± SEM. *N* = 5 mice shCtrl (49 nuclei), *N* = 4 mice sh1-Suv39h1 (29 nuclei), *N* = 3 mice sh2-Suv39h2 (46 nuclei). **(F–N)** Retroviruses expressing shCtrl, sh1-Suv39h1 or sh2-Suv39h2 and the fluorescent protein ZsGreen (ZsG) were injected into the dentate gyrus of 2-month-old mice by stereotaxic surgery. Animals were sacrificed 1 week after retrovirus injection. **(F)** Immunofluorescence staining of DCX and GFAP in the dentate gyrus of mice injected with the retroviruses. Nuclei were stained with NucBlue (NucB). Arrows indicate ZsG+DCX+ cells, arrowheads indicate ZsG+DCX- cells. Scale bars: 20 μm. Panels to the right show separated channels of a section of the images shown at the left. **(G)** Quantification of the percentage of DCX+GFAP- cells of the total number of ZsG+ cells in the GCL. **(H)** Representative ZsG+GFAP+DCX- cell with radial glia-like (RGL) morphology. **(I, J)** Quantification of the percentage of GFAP+DCX- cells of the total number of ZsG+ cells with RGL **(I)** or astrocyte **(J)** morphology. **(K)** Representative ZsG+ cells Sox2+DCX- (Type 2a), Sox2+DCX+ (Type 2b), Sox2-DCX+ (neuroblast). Nuclei were stained with NucB. Scale bars: 20 μm. Panels to the right show separated channels of cells indicated with an arrow at the left images. **(L–N)** Quantification of the percentage of Sox2+DCX- **(L)**, Sox2+DCX+ **(M)**, Sox2-DCX+ **(N)** cells of the total number of ZsG+ cells. Data are presented as mean ± SEM; *N* = 5 animals **(E–H, J–L)**. *p < 0.05, **p < 0.01, ****p < 0.0001, one-way ANOVA followed by Bonferroni posthoc test. GCL: Granule cell layer; SGZ: Subgranular zone; H: hilus.

Then, AHPs were transduced with retroviruses expressing the selected shRNAs. Compared to cells trasduced with shCtrl, a significant decrease in Suv39h1 and Suv39h2 mRNA (Figures 7A,B) and protein (Figure 7C) levels was observed in cells transduced with retroviruses expressing sh1-Suv39h1 and sh2-Suv39h2, respectively. To assess the effect of Suv39h1/h2 knockdown on the generation of new neurons *in vivo*, the retroviruses encoding for shCtrl, sh1-Suv39h1 or sh2-Suv39h2 were stereotaxically injected into the dorsal and ventral DG of 2-month-old mice, and mice were were euthanized 1 week post injection (wpi). Retrovirus only transduce proliferating cells and are passed on their progeny. No significant changes in the number of cells positive for active caspase 3 were observed between mice injected with the different retroviruses, or among ipsi- and contralateral DG (Supplementary Figures S8A,B), suggesting that retroviral transduction or Suv39h1 and Suv39h2 knockdown did not induce cell apoptosis. As expected, a reduction in H3K9me3 levels was observed in Suv39h1 and Suv39h2 deficient cells (Figures 7D,E). To assess neuronal differentiation, the percentage of shRNA-expressing cells (positive for ZsG, ZsG+, Supplementary Figure S8C) that express DCX but not GFAP (Figure 7F, arrows) was evaluated. A significant decrease in the percentage of ZsG+ cells DCX+ GFAP- was observed in mice transduced with sh1-Suv39h1 or sh2-Suv39h2 compared to control mice (Figure 7G). No changes in the percentage of ZsG+ cells expressing GFAP with radial glia-like (Figures 7H,I) or astrocyte (Figure 7J) morphology were observed between Suv39h1/h2-deficient cells compared to control cells expressing shCtr. These results indicate that Suv39h1 and Suv39h2 knockdown impairs the generation of new neurons while not affecting the NSC pool or the generation of astrocytes.

Finally, we evaluated the percentage of ZsG+ NPCs (Figure 7K). Type 2 NPCs are classified as type 2a or type 2b based on their neuronal commitment; type 2a are positive for Sox2 and negative for DCX (Sox2+DCX-), while type 2b are positive for both markers (Sox2+DCX+) (Kempermann et al., 2004). In mice transduced with sh1-Suv39h1 or sh2-Suv39h2, a significant increase in the percentage of ZsG+ cells Sox2+ DCX- was observed (Figure 7L), while the percentage of ZsG+ cells that were Sox2+DCX+ (Figure 7M) was significantly reduced. A decrease in the percentage of ZsG+ neuroblasts and immature neurons Sox2-DCX+ was observed (Figure 7N), in agreement with Figure 7F. Altogether, the increase in type 2a cells and decrease in type 2b and neuroblasts/immature neurons, suggest that Suv39h1- and Suv39h2-deficient type 2a cells are unable to differentiate into type 2b cells that can develop into neuroblasts and subsequently into granule neurons.

## Discussion

The generation of new neurons in the adult hippocampus is a highly regulated process that is controlled by extrinsic and intrinsic factors. In the present study, we have explored the involvement of the repressive modification H3K9me3 and the enzymes that introduce this modification in adult hippocampal neurogenesis. First, we characterized in the adult mouse DG the expression and distribution of H3K9me3 during the stages of neurogenesis by *in situ* analysis using a specific antibody that allows analyzing global changes in this modification (Liu et al., 2015; Calderon-Garciduenas et al., 2020; Jury et al., 2020). Using this approach, we determined a transient increase of H3K9me3 staining in NPCs and neuroblasts compared to NSCs, that decreased towards neuronal maturation. Interestingly, in embryonic stem cells (ESCs) chromatin reorganizes globally as these cells differentiate into NPCs, losing their pluripotency (Meshorer et al., 2006), and an expansion of H3K9me3 domains is observed in differentiated cells compared to human ESCs (Hawkins et al., 2010; Becker et al., 2016). In agreement, we determined an increase in H3K9me3 foci in NPCs compared with NSCs, which showed a diffuse nuclear distribution of H3K9me3, and in neuroblasts there was a strong increase in the number of small H3K9me3 foci that were co-distributed with NucB-chromocenters. This increase in small H3K9me3 foci could be related to the beginning of global chromatin reorganization when differentiation initiates (Meshorer et al., 2006). In line with our results, in the mouse and baboon subventricular zone (SVZ), another neurogenic niche of the postnatal brain, H3K9me3 staining was found enriched in NSCs and neuroblasts, and decreases after differentiation (Foret et al., 2014). Concomitantly with the increase in H3K9me3 staining we observed an increase in H3K9me2 foci and H3K9me1 staining intensity, suggesting global changes in H3K9 methylation that might contribute to chromatin reorganization during differentiation.

The strong increase in H3K9me3 foci at early stages of adult neurogenesis suggests that H3K9me3 heterochromatin might play a role in silencing stemness genes required to allow neuronal differentiation. In line with this notion, H3K9me3 plays a critical role in marking chromatin to silence stem/memory genes during murine CD8^+^T cells terminal differentiation (Pace et al., 2018). In addition, in neuroblasts, H3K9me3 may also mediate the repression of lineage-inappropriate genes as previously demonstrated in different cell types, where the H3K9me3 mark increases during differentiation (Allan et al., 2012; Liu et al., 2015; Nicetto and Zaret, 2019).

The relevance of H3K9me3 in neuronal differentiation is supported by the results with the pharmacological inhibition of the H3K9 methyltransferases Suv39h1 and Suv39h2, showing that chaetocin reduced differentiation of AHPs. These results are the first to demonstrate that an epidrug can impact the neurogenic process. Among H3K9 methyltransferases evaluated, the enzymes Suv39h1 and Suv39h2 exhibited a similar pattern of expression than H3K9me3/me2 in cultured progenitors with high levels of expression at early stages of neurogenesis that decreased upon differentiation. Interestingly, Suv39h1 protein levels decreased dramatically upon differentiation, prior to Suv39h1 lowering the mRNA levels. These early changes in Suv39h1 protein levels could be mediated by post-translation modifications as occurs during development, where in addition to the transcriptional repression of Suv39h1 there is a proteasomal degradation of this enzyme involved to maintain low levels of H3K9me3 in specific genomic loci (Zhang et al., 2018; Kim et al., 2021).

The involvement of H3K9me3 in differentiation was also demonstrated by our *in vivo* findings when knocking down the expression of Suv39h1 and Suv39h2. Moreover, reduced levels of H3K9me3 were observed when Suv39h1 or Suv39h2 were knocked down *in vitro* and *in vivo*, indicating that these enzymes introduce H3K9me3 in hippocampal neural precursor cells. This agrees with evidence indicating that Suv39h enzymes are the major methyltransferases for pericentric H3K9 methylation (Peters et al., 2001). *In vivo* knockdown of Suv39h1 and Suv39h2 in the adult mouse hippocampus induced a decrease in type 2b cells, neuroblast and immature neurons, while inducing an increase in type 2a progenitors. These results suggest that Suv39h1/h2-mediated H3K9 methylation is critical for neuronal differentiation of NPCs in the adult DG.

The increase in H3K9me3 foci during early stages of neurogenesis could also be associated with the cell cycle exit of NPCs and neuroblasts, as observed in other cell types. For example, in adult cardiomyocytes H3K9me3 is strongly required for the cell cycle exit, and loss of H3K9me3 leads to increased cell cycle gene expression resulting in enhanced cardiomyocyte proliferation (El-Nachef et al., 2018). Also, ChIP-seq analysis in purified SVZ cells from baboon brain revealed that H3K9me3 is enriched for genes involved in the cell cycle network, proliferation, CNS fate commitment, among others (Foret et al., 2014). An integrated ChIP-Seq and RNA-Seq analysis furthermore suggested that H3K9me3 is important for the maintenance of cell identity to prevent improper lineage differentiation (Foret et al., 2014). In agreement, we determined in cultured AHPs that pharmacological inhibition of Suv39h1 and Suv39h2 induced proliferation. Therefore, Suv39h1/h2-mediated H3K9 methylation might repress proliferation genes and contribute to progenitor cell differentiation and neurogenesis.

Interestingly, immunodetection of H3K9me3 in mature neurons revealed a decrease in the number but an increase in the size of H3K9me3 foci that co-localize with NucB chromocenters. This suggests that upon neuronal maturation H3K9me3 is redistributed to cHC. This evidence agrees with studies showing that once lineage-commitment is acquired and after completion of maturation H3K9me3 is mostly present in the cHC (Ait-Si-Ali et al., 2004; Guasconi et al., 2010; Nicetto and Zaret, 2019). Moreover, Suv39h1 knockdown inhibits differentiation and maturation of myotubes concomitantly with the loss of H3K9me3 in the pericentromeric heterochromatin (Ait-Si-Ali et al., 2004). We could not evaluate the potential effect of Suv39h1/h2 knockdown on mature newborn neurons since Suv39h1/h2-deficient progenitors were unable to differentiate into neurons.

In summary, we have determined for the first time that during adult hippocampal neurogenesis there are global changes in H3K9 methylation that are crucial for the neurogenic process, and established that the H3K9 methyltransferases Suv39h1 and Suv39h2 control the differentiation of NPCs during neurogenesis. Further analysis including ChIPseq will be needed to assess specific genes controlled by the H3K9me3 modification during the progression of neurogenesis. Since epigenetic mechanisms are the interface between genes and the environment (Jaenisch and Bird, 2003; Zhang and Meaney, 2010), H3K9 methylation might contribute to the regulation of hippocampal neurogenesis by environmental and physiological stimuli. In addition, H3K9me2/me3 loss in the hippocampus has been observed in aging (Rodriguez-Matellan et al., 2020) and neurodegenerative diseases (Frost et al., 2014; Hernandez-Ortega et al., 2016; Mansuroglu et al., 2016; Jury et al., 2020), suggesting that it may contribute to neurogenesis impairment in these conditions. It will be interesting to evaluate whether increasing H3K9me2/me3 levels (e.g., by overexpression of Suv39h1 and Suv39h2) would be able to restore neurogenesis in these conditions.

Our findings contribute to the comprehension of the epigenetic mechanisms controlling adult hippocampal neurogenesis, which might be relevant for future therapeutic strategies aimed to stimulate this process.

## Data Availability

The raw data supporting the conclusion of this article will be made available by the authors, without undue reservation.
