# Supplementary material for: H3K9 Methyltransferases Suv39h1 and Suv39h2 Control the Differentiation of Neural Progenitor Cells in the Adult Hippocampus
Source: Front Cell Dev Biol. 2022 Jan 12;9:778345. doi: 10.3389/fcell.2021.778345 (PMC8791356; doi:10.3389/fcell.2021.778345)
Supplement: Supplementary file 1 [file DataSheet1.PDF]

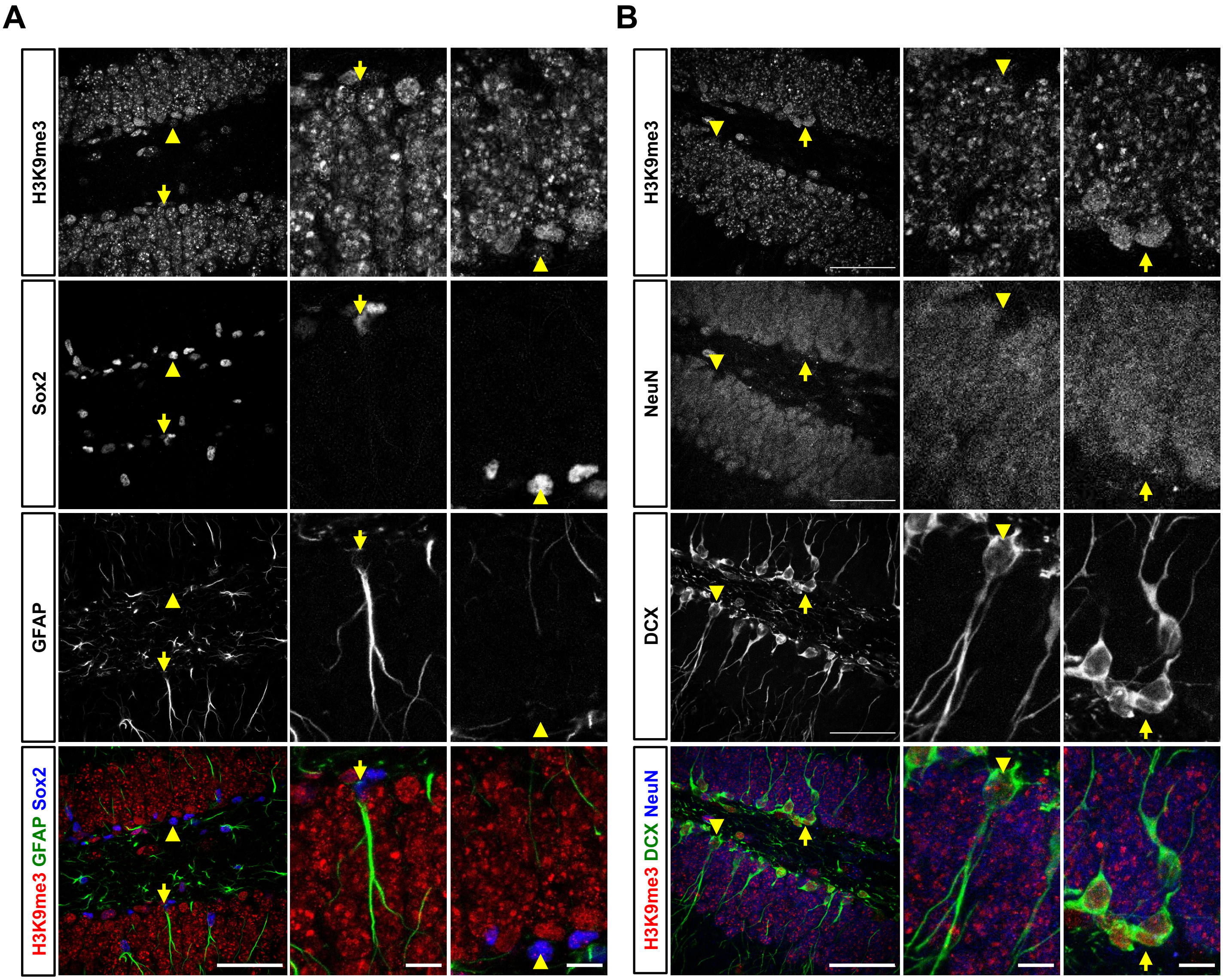

Supplementary Figure S1. H3K9me3 staining during the stages of neurogenesis in the adult mouse hippocampus. (A) Immunostaining of H3K9me3, GFAP and Sox2 in the DG of 2-month-old mouse. The arrow indicates a NSC (GFAP+Sox2+), the arrowhead indicates a NPC (GFAP-Sox2+). Scale bar: 50 µm. Panels to the right show higher magnification images. Scale bar 10 µm. **(B)** Immunofluorescence staining of H3K9me3, DCX and NeuN in the DG. Arrow indicates a neuroblast and the arrowhead indicates an immature neuron. Scale bar 50 µm. Panels to the right show higher magnification images. Scale bar 10 µm.

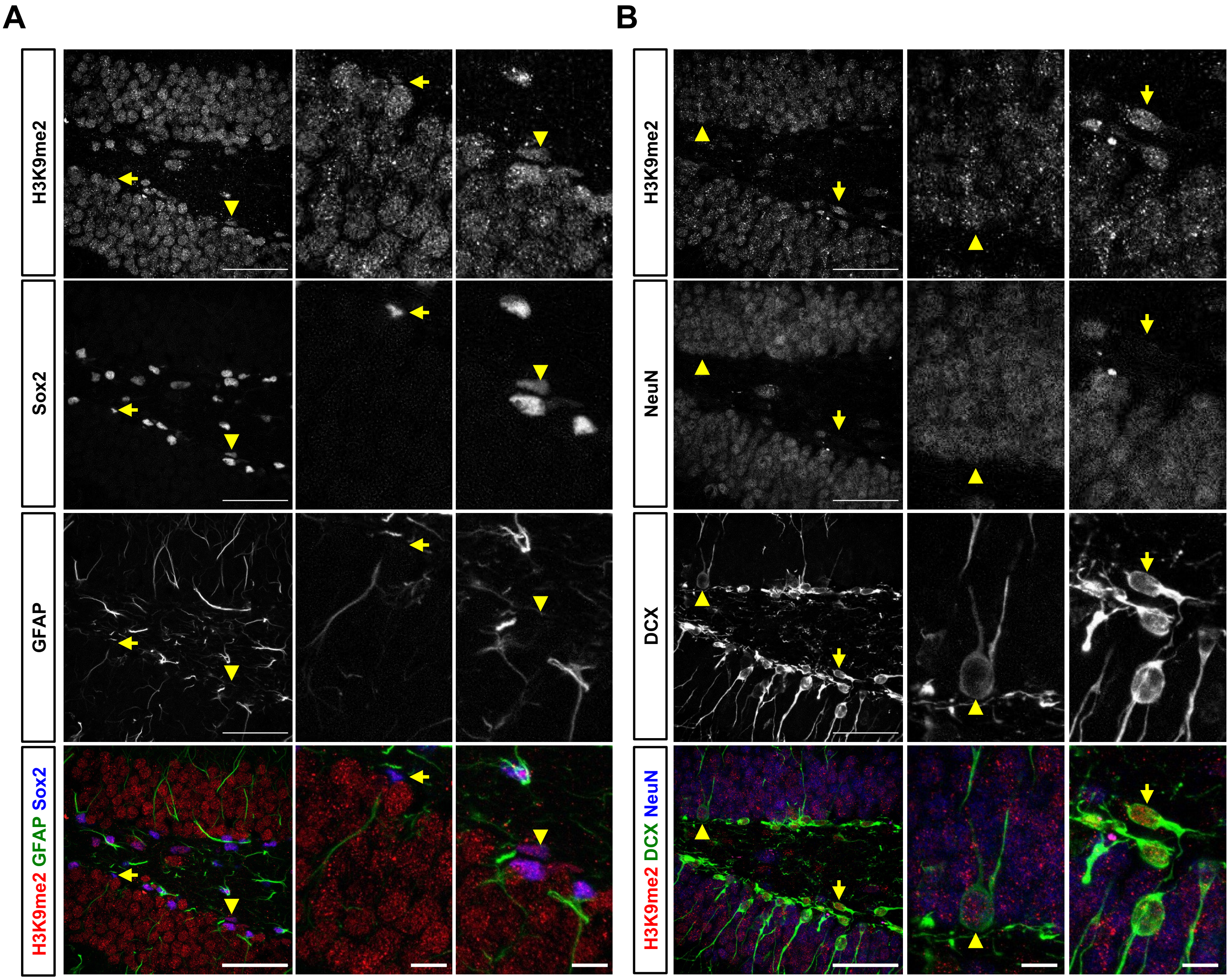

Supplementary Figure S2. H3K9me2 staining during the stages of neurogenesis in the adult mouse hippocampus. (A) Immunostaining of H3K9me2, GFAP and Sox2 in the DG of 2-month-old mouse. The arrow indicates a NSC (GFAP+Sox2+), the arrowhead indicates a NPC (GFAP-Sox2+). Scale bar: 50 µm. Panels to the right show higher magnification images. Scale bar 10 µm. **(B)** Immunofluorescence staining of H3K9me2, DCX and NeuN in the DG. Arrow indicates a neuroblast and the arrowhead indicates an immature neuron. Scale bar 50 µm. Panels to the right show higher magnification images. Scale bar 10 µm.

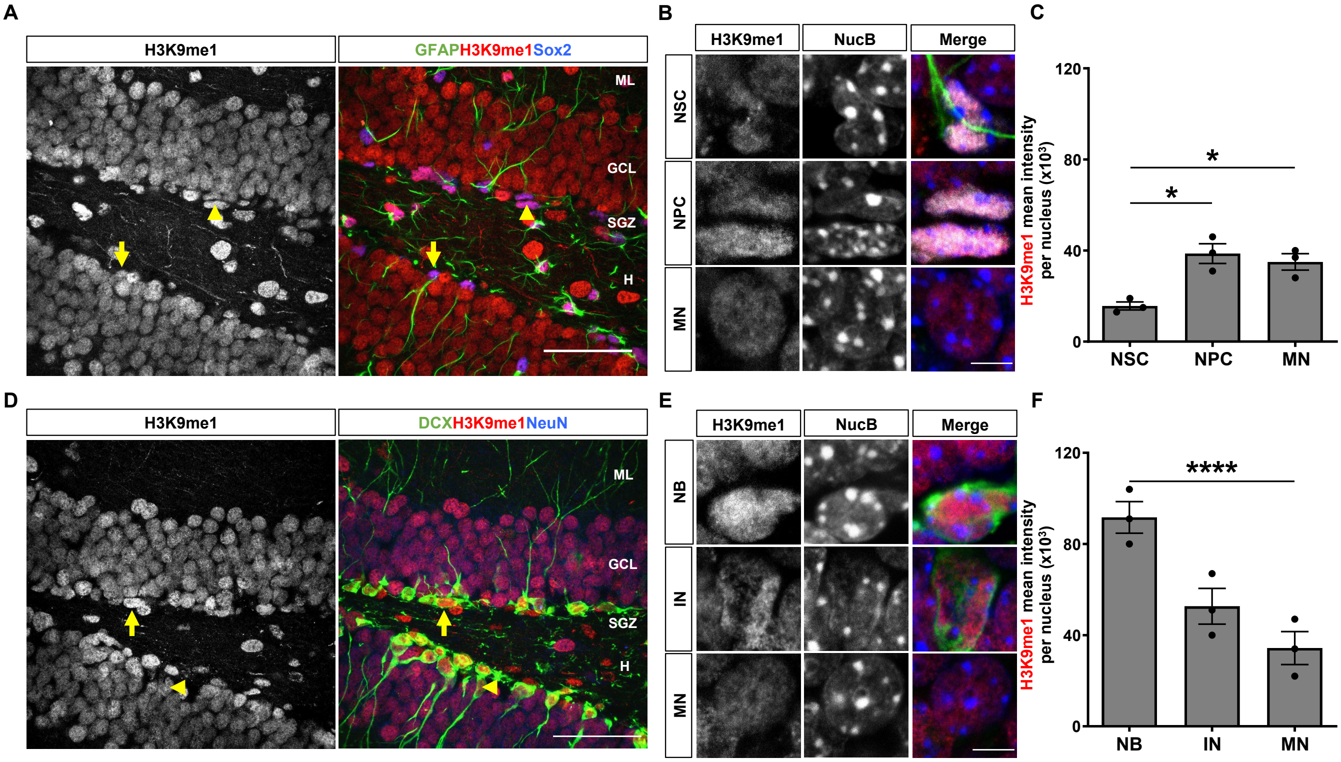

Supplementary Figure S3. H3K9me1 staining during the stages of neurogenesis in the adult mouse hippocampus. **(A)** Immunostaining of H3K9me1, GFAP and Sox2 in the DG of 2-month-old mouse. The arrow indicates a NSC (GFAP+Sox2+), the arrowhead indicates an NPC (GFAP-Sox2+). Scale bar 50 μm. **(B)** Digital zoom of nuclei from NSC (top), NPC (middle) and mature neuron (bottom). GFAP (green), H3K9me1 (red), Sox2 (white) NucB (blue). Scale bar 10 μm. **(C)** Mean intensity of H3K9me1 staining per nucleus. **(D)** Immunofluorescence staining of H3K9me1, DCX and NeuN in the DG. Arrow indicates a neuroblast and the arrowhead indicates an immature neuron. Scale bar 50 μm. **(E)** Digital zoom of nuclei from neuroblast (top), immature neuron (middle) and mature neuron (bottom). DCX (green), H3K9me1 (red), NucB (blue). **(F)** Mean intensity of H3K9me1 staining per nucleus Bars represent mean ± SEM. *p<0.05; ****p<0.0001. One-way ANOVA followed by Bonferroni posthoc test, N=3 mice. ML: Molecular layer; GCL: Granule cell layer; SGZ: Subgranular zone, H: Hilus.

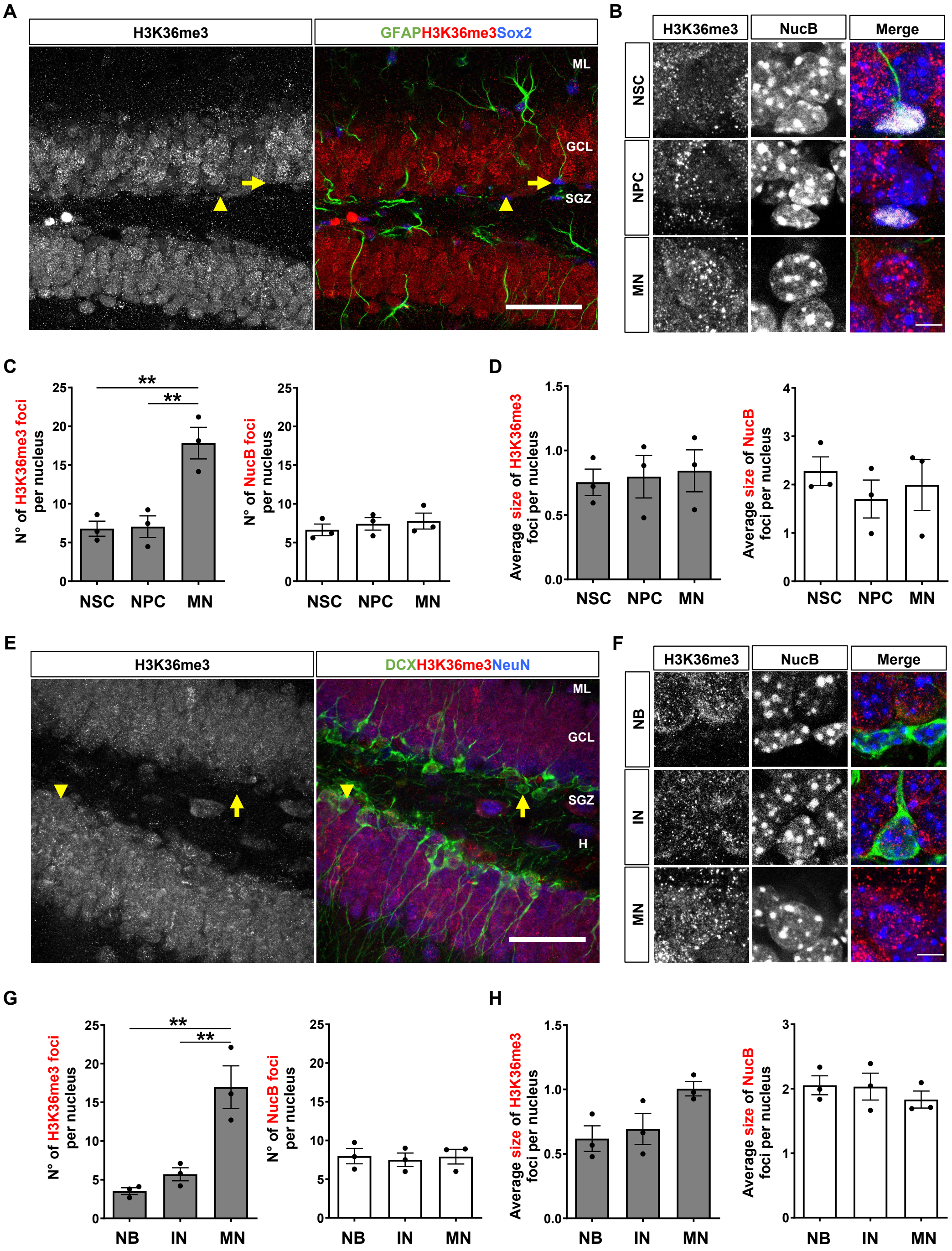

Supplementary Figure S4. H3K36me3 staining during the stages of neurogenesis in the adult mouse hippocampus. (A) Immunostaining of H3K36me3, GFAP and Sox2 in the DG of 2-month-old mouse. The arrow indicates a NSC (GFAP+Sox2+), the arrowhead indicates a NPC (GFAP-Sox2+). Scale bar 50 µm. **(B)** Digital zoom of nuclei from NSC (top), NPC (middle) and mature neuron (bottom). GFAP (green), H3K36me3 (red), Sox2 (white) NucB (blue). Scale bar 10 µm. **(C)** Average number of H3K36me3 and NucB foci per nucleus of NSC, NPC and mature neurons (MN). **(D)** Average size of H3K36me3 and NucB foci in the different cell types. **(E)** Immunofluorescence staining of H3K36me3, DCX and NeuN in the DG. Arrow indicates a neuroblast and the arrowhead indicates an immature neuron. Scale bar 50 µm. **(F)** Digital zoom of nuclei from neuroblast (top), immature neuron (middle) and mature neuron (bottom). **(G)** Average number of H3K36me3 and NucB foci per nucleus of neuroblasts (NB), immature neurons (IN) and mature neurons (MN). **(H)** Average size of H3K36me3 and NucB foci in the different cell types. Bars represent mean ± SEM. $^{**}p<0.01$. One-way ANOVA followed by Bonferroni posthoc test, N=3 mice. ML: Molecular layer; GCL: Granule cell layer; SGZ: Subgranular zone, H: Hilus.

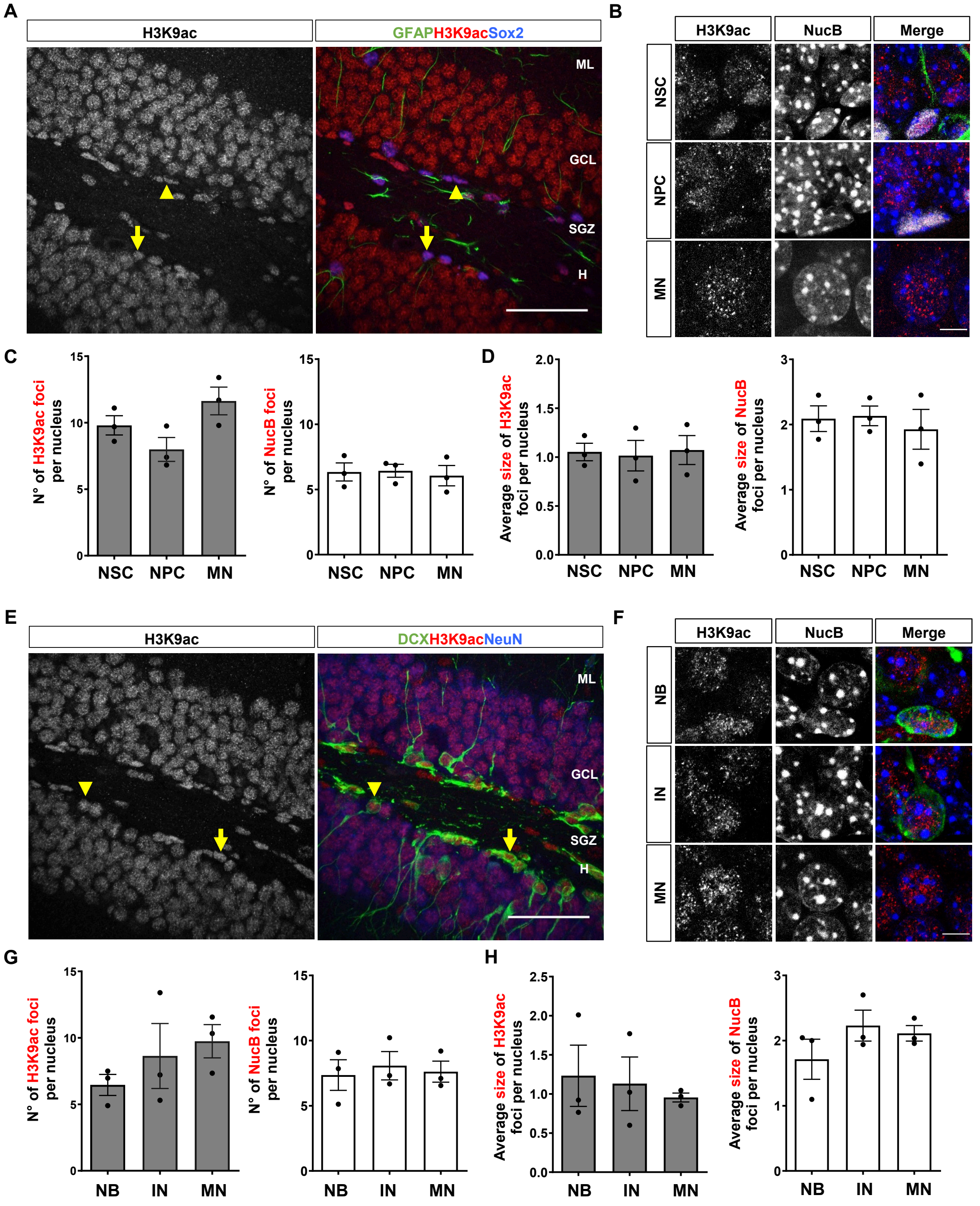

Supplementary Figure S5. H3K9ac staining during the stages of neurogenesis in the adult mouse hippocampus. (A) Immunostaining of H3K9ac, GFAP and Sox2 in the DG of 2-month-old mouse. The arrow indicates a NSC (GFAP+Sox2+), the arrowhead indicates a NPC (GFAP-Sox2+). Scale bar 50 µm. **(B)** Digital zoom of nuclei from NSC (top), NPC (middle) and mature neuron (bottom). GFAP (green), H3K9ac (red), Sox2 (white) NucB (blue). Scale bar 10 µm. **(C)** Average number of H3K9ac and NucB foci per nucleus of NSC, NPC and mature neurons (MN). **(D)** Average size of H3K9ac and NucB foci in the different cell types. **(E)** Immunofluorescence staining of H3K9ac, DCX and NeuN in the DG. Arrow indicates a neuroblast and the arrowhead indicates an immature neuron. Scale bar 50 µm. **(F)** Digital zoom of nuclei from neuroblast (top), immature neuron (middle) and mature neuron (bottom). **(G)** Average number of H3K9ac and NucB foci per nucleus of neuroblasts (NB), immature neurons (IN) and mature neurons (MN). **(H)** Average size of H3K9ac and NucB foci in the different cell types. Bars represent mean±SEM. Not significant differences were observed, One-way ANOVA followed by Bonferroni posthoc test, N=3 mice. ML: Molecular layer; GCL: Granule cell layer; SGZ: Subgranular zone, H: Hilus.

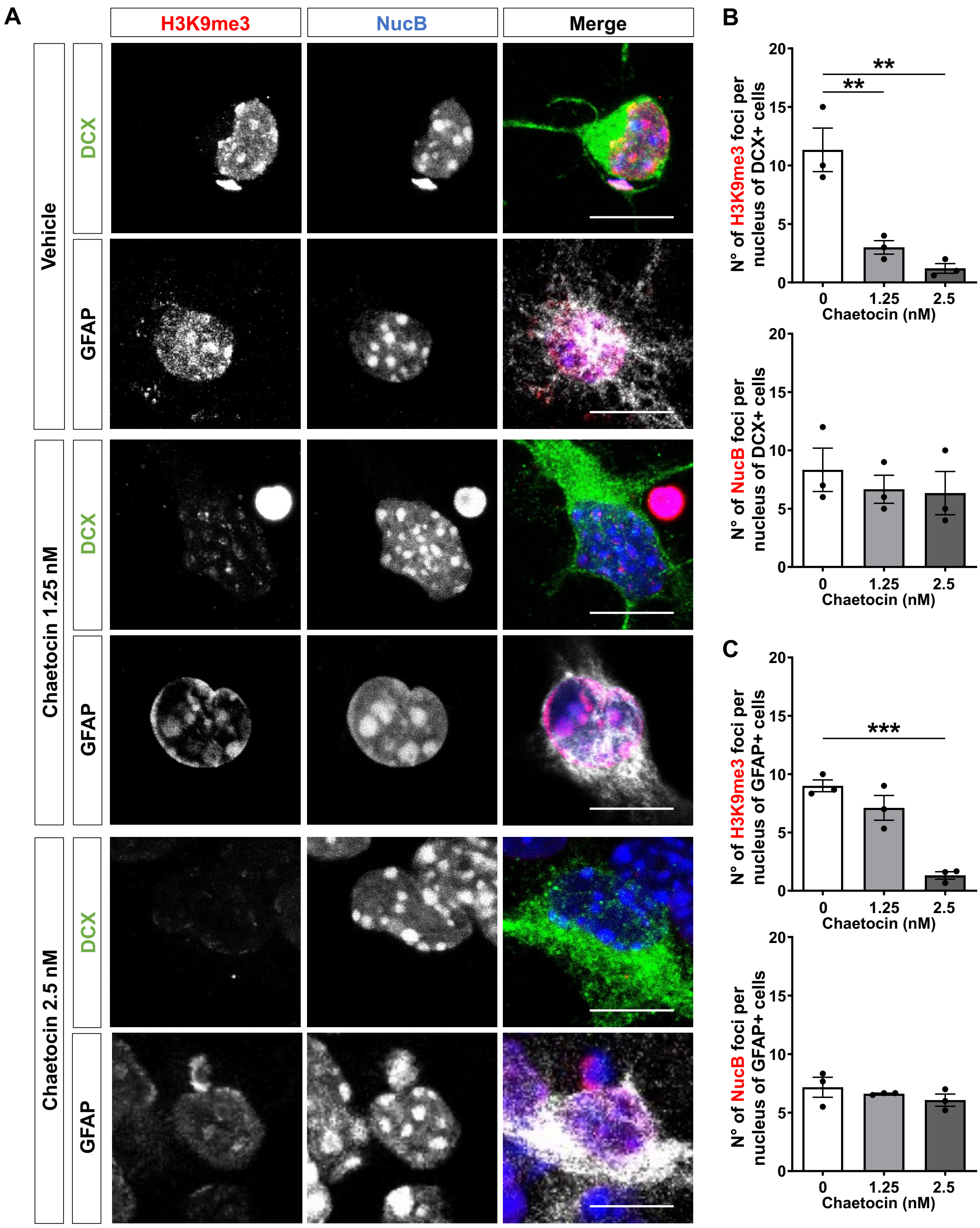

Supplementary Figure S6. Chaetocin reduces the number of H3K9me3 foci cultured adult hippocampal progenitors. (A) Immunostaining of H3K9me3 (red), DCX (green) and GFAP (white) in AHPs differentiated for 24 h in the presence or absence of 1.25 and 2.5 nM chaetocin. NucB (blue) was used as nuclear staining. Scale bar: 10 µm. **(B, C)** Average number of H3K9me3 and NucB foci per nucleus AHP-derived neurons (B) and astrocytes (C). Bars represent mean±SEM. **$p<0.01$, ***$p<0.001$. One-way ANOVA followed by Bonferroni posthoc test, N=3 independent experiments.

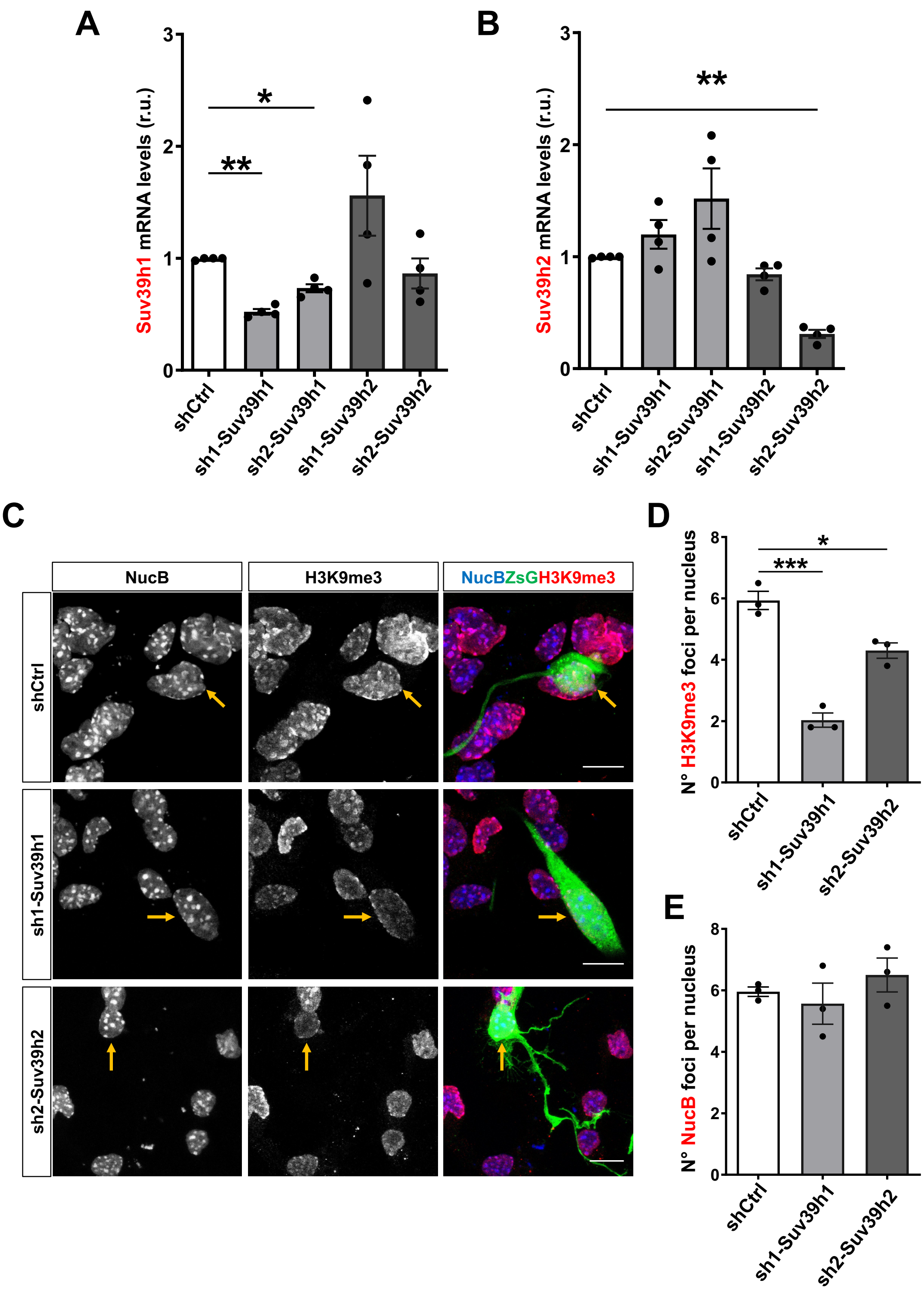

Supplementary Figure S7. Suv39h1 and Suv39h2 knockdown in AHP. (A, B) RT-qPCR from total RNA isolated from AHP 48 hours post-transfection with a control shRNA (shCtrl) or shRNAs targeting Suv39h1 (sh1-Suv39h1 and sh2-Suv39h1) or Suv39h2 (sh1-Suv39h2 and sh2-Suv39h2). Suv39h1 (A) and Suv39h2 (B), were normalized to GAPDH mRNA and expressed relative to shCtrl. **(C)** Immunostaining of H3K9me3 (red) and ZsG (green) in AHP 48 hours post-transfection with sh-Ctrl, sh1-Suv39h1 and sh2-Suv39h2. Scale bar: 20 µm . **(D, E)** Average number of H3K9me3 (D) and NucB (E) foci per nucleus in AHP 48 hours post-transfection with sh-Ctrl, sh1-Suv39h1 and sh2-Suv39h2. Bars represent mean±SEM. *p<0.05, **p<0.01, ***p<0.001. One-way ANOVA followed by Bonferroni posthoc test, N=4 (A,B) or N=3 (D,E) independent experiments.

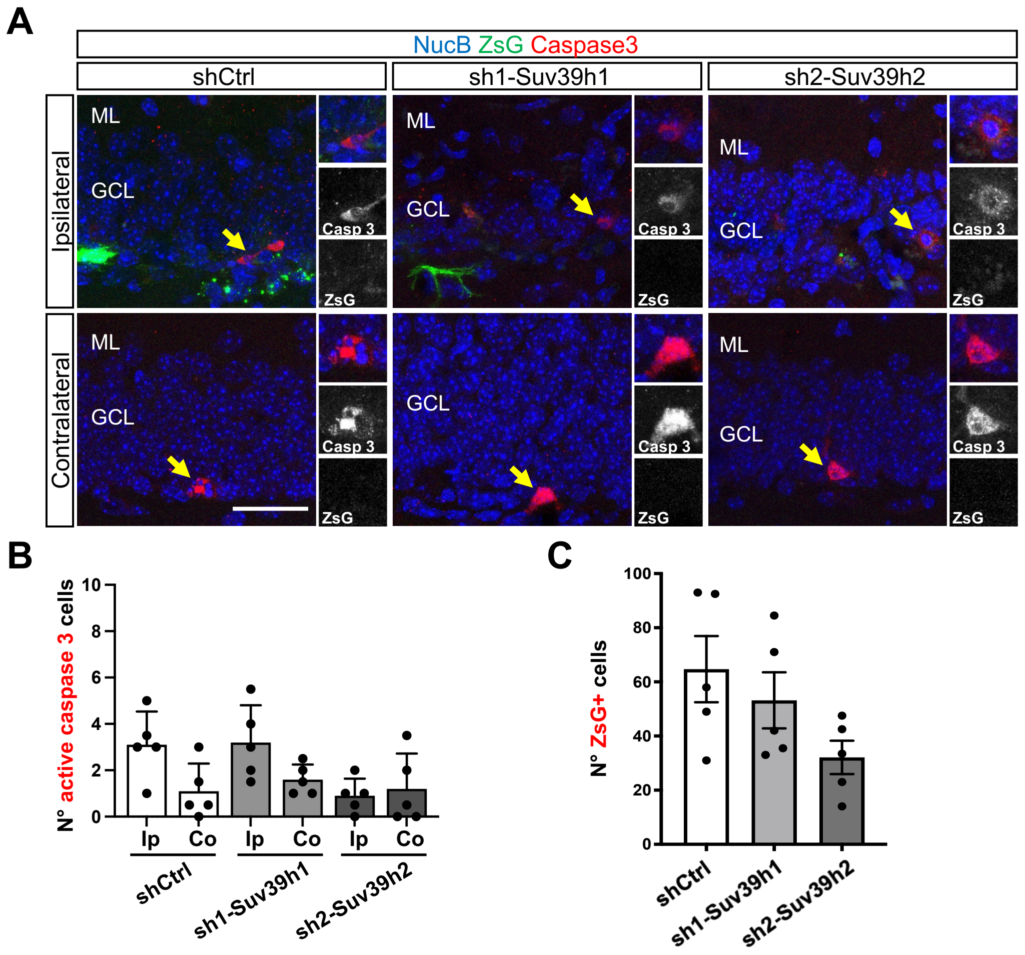

Supplementary Figure S8. Active caspase 3 detection and number of ZsG positive cells in the dentate gyrus of mice injected with shRNA expressing retroviruses. (A) Immunostaining of active caspase 3 (red) and ZsG (green) in the dentate gyrus 1 week post injection of retroviruses expressing a control shRNA (shCtrl) or shRNAs targeting Suv39h1 (sh1-Suv39h1) or Suv39h2 (sh2-Suv39h2). NucB (blue) was used as nuclear staining. Scale bar: 30 µm. Images at the right show higher magnification of separated channels of cells positive for active caspase 3 (arrow). No ZsG+ cells positive for active Caspase 3 were found. **(B)** Graph shows mean number of active caspase 3 cells per tissue section. Data are presented as mean ± SEM; N=5 mice. No statistical differences were observed between experimental groups or between ipsilateral (Ip) and contralateral (Co), one-way ANOVA followed by Bonferroni posthoc test. GCL: Granule cell layer; ML: Molecular layer. **(C)** Total number of shRNA-expressing cells (positive for ZsG, ZsG+) in one set of tissue sections. Data are presented as mean ± SEM; N=5 mice. Not significant differences were observed; one-way ANOVA followed by Bonferroni posthoc test.
